# Low-Volume Bowel Preparation Is Associated With Reduced Time to Colonoscopy in Hospitalized Patients: A Propensity-Matched Analysis

**DOI:** 10.14309/ctg.0000000000000482

**Published:** 2022-03-28

**Authors:** Christopher L.F. Sun, Darrick K. Li, Ana Cecilia Zenteno, Marjory A. Bravard, Peter Carolan, Bethany Daily, Sami Elamin, Jasmine Ha, Amber Moore, Kyan Safavi, Brian J. Yun, Peter Dunn, Retsef Levi, James M. Richter

**Affiliations:** 1 Sloan School of Management, Massachusetts Institute of Technology, Cambridge, Massachusetts, USA;; 2 Healthcare Systems Engineering, Massachusetts General Hospital, Boston, Massachusetts, USA;; 3 Section of Digestive Diseases, Department of Medicine, Yale School of Medicine, New Haven, Connecticut, USA;; 4 Perioperative Services, Massachusetts General Hospital, Boston, Massachusetts, USA;; 5 Harvard Medical School, Harvard, Boston, Massachusetts, USA;; 6 Division of General Internal Medicine, Department of Medicine, Massachusetts General Hospital, Boston, Massachusetts, USA;; 7 Gastrointestinal Division, Massachusetts General Hospital, Boston, Massachusetts, USA;; 8 Department of Emergency Medicine, Massachusetts General Hospital, Boston, Massachusetts, USA.

## Abstract

**INTRODUCTION:**

Delays in inpatient colonoscopy are commonly caused by inadequate bowel preparation and result in increased hospital length of stay (LOS) and healthcare costs. Low-volume bowel preparation (LV-BP; *sodium sulfate, potassium sulfate, and magnesium sulfate*) has been shown to improve outpatient bowel preparation quality compared with standard high-volume bowel preparations (HV-BP; *polyethylene glycol*). However, its efficacy in hospitalized patients has not been well-studied. We assessed the impact of LV-BP on time to colonoscopy, hospital LOS, and bowel preparation quality among inpatients.

**METHODS:**

We performed a propensity score-matched analysis of adult inpatients undergoing colonoscopy who received either LV-BP or HV-BP before colonoscopy at a quaternary academic medical center. Multivariate regression models with feature selection were developed to assess the association between LV-BP and study outcomes.

**RESULTS:**

Among 1,807 inpatients included in this study, 293 and 1,514 patients received LV-BP and HV-BP, respectively. Among the propensity score-matched population, LV-BP was associated with a shorter time to colonoscopy (β: −0.43 [95% confidence interval: −0.56 to −0.30]) while having similar odds of adequate preparation (odds ratio: 1.02 [95% confidence interval: 0.71–1.46]; *P* = 0.92). LV-BP was also significantly associated with decreased hospital LOS among older patients (age ≥ 75 years), patients with chronic kidney disease, and patients who were hospitalized with gastrointestinal bleeding.

**DISCUSSION:**

LV-BP is associated with decreased time to colonoscopy in hospitalized patients. Older inpatients, inpatients with chronic kidney disease, and inpatients with gastrointestinal bleeding may particularly benefit from LV-BP. Prospective studies are needed to further establish the role of LV-BP for inpatient colonoscopies.

## INTRODUCTION

Adequate bowel cleansing is a critical component in the care of hospitalized patients undergoing diagnostic or therapeutic colonoscopy. Inadequate bowel preparation is associated with worse clinical outcomes, including increased rates of complications, missed pathologic lesions, repeated bowel preparation medication administration, and even aborted colonoscopy procedures (1–6). In turn, this can result in increased hospital length of stay (LOS) and healthcare costs (1,7). An estimated 30%–50% of inpatients undergoing colonoscopy suffer from inadequate colon cleansing (8,9). As such, significant healthcare resources are expended by delays stemming from poor preparation. Moreover, few strategies have been rigorously shown to reduce the risk of inadequate bowel preparation and delays in inpatient colonoscopy (10–12).

Low-volume bowel preparations (LV-BPs), such as *sodium sulfate, potassium sulfate, and magnesium sulfate* (Suprep), have been found to increase preparation quality and tolerability in outpatients, representing an attractive alternative to the standard high-volume *polyethylene glycol* (PEG) preparations (13,14). However, it is unknown whether LV-BPs will be similarly beneficial in the bowel preparation of hospitalized patients, particularly given the substantial clinical differences in the outpatient and inpatient populations, including increased medical acuity and comorbidities in the latter group (15,16). As such, determining the efficacy of LV-BPs in hospitalized patients is important to determine whether LV-BPs represent a new strategy to reduce delays to endoscopy, hospital LOS, and healthcare costs.

The aim of this study was to evaluate the impact of LV-BP on time to colonoscopy, quality of bowel preparation, and hospital LOS in hospitalized patients as compared with a propensity score-matched (PSM) historical cohort who received traditional high-volume PEG-based preparation.

## METHODS

### Study design and population

This single-center, retrospective, observational study, performed at a quaternary academic medical center, compared the differences between low-volume and high-volume bowel preparations for inpatient colonoscopies. Hospitalized adult patients (18 years or older) from January 1, 2018, to January 31, 2021, undergoing colonoscopy at this center were included in this study. Patients who had ileus, toxic megacolon, evidence of gastrointestinal obstruction, an allergy to either bowel preparation, received bowel preparation for other reasons before their colonoscopy, or who were unable to give consent to the procedure were excluded from the analysis.

### Bowel preparation and endoscopy

The suitability of the patient for colonoscopy was determined by the inpatient gastroenterology consult team, consisting of fellows and an attending gastroenterologist. Patients received either traditional high-volume PEG-based bowel preparation (GoLYTELY, referred to as HV-BP) or low-volume bowel preparation containing sodium sulfate, potassium sulfate, and magnesium sulfate (Suprep, referred to as LV-BP). LV-BP became available starting on June 16, 2020, and became the primary bowel preparation medication ordered by inpatient providers. HV-BP could still be used at the discretion of the consulting team based on underlying patient comorbidities (e.g., chronic kidney disease). Both bowel preparations were administered using split-dose administration (half volume administered the evening before the procedure and half volume the morning of the procedure). If the colon preparation was still incomplete after this, an additional split dose was given to the patient.

Colonoscopies were performed in the endoscopy unit at Massachusetts General Hospital. The Massachusetts General Hospital endoscopy unit has 1 procedure room dedicated each day for inpatient procedures alone and is also open for procedures on both weekend days. One anesthesiologist supervises the certified registered nurse anesthetist assigned to the inpatient endoscopy room. Procedures were performed under moderate sedation (midazolam and fentanyl) or deep sedation (propofol, midazolam, and fentanyl) at the discretion of the gastroenterologist and anesthesia providers.

The fellow performed the colonoscopy under the direct supervision of an attending gastroenterologist. Procedure notes were documented in Provation, an endoscopy procedure documentation software, by the fellow and reviewed by the attending. All colonoscopies were performed using Olympus 190-series (CF-HQ190AL and PCF-H190L) colonoscopes. The success of cecal intubation was established by visualization of the appendiceal orifice and ileocecal valve.

### Data sources and collection

Baseline patient characteristics, hospitalization characteristics, and study outcomes were obtained through patient electronic health records. Patient hospitalization encounters were categorized using a Medicare severity diagnosis-related group and as *International Classification of Disease, 10th revision* codes (17). Baseline results were defined as the first measurement taken at the hospital. Relative differences in laboratory results before and after the colonoscopy procedure were also calculated. To account for changes in patient and hospitalization characteristics due to the COVID-19 pandemic, hospitalizations occurring between March 15 to June 8, 2020, corresponding to when Boston's COVID-19 public health advisories were issued and relaxed, respectively, were identified as such and controlled for in each regression model.

### Study outcomes

The primary outcome of this study was time to colonoscopy. This was defined as the time from when the first bowel preparation order was administered to the patient to the colonoscopy procedure. This duration of time was measured in days to minimize the impact of heterogenous patient characteristics, including inpatient admission time and time of the colonoscopy procedure, that may artificially change the duration of time had it been measured in hours. The secondary outcomes were hospital LOS, defined as the duration from inpatient admission decision to hospital discharge, and adequate bowel preparation, defined as an Aronchick score of 1 or 2 (“Excellent” or “Good”) (18). Additional quality measures including requirement for an additional bowel preparation medication order (i.e., when an additional [more than one] bowel preparation medication order of the same medication type was needed to complete preparation), polyp detection rate, in-hospital mortality, physician-reported procedure complications, and use of adjunctive laxatives for bowel preparation (i.e., enemas, magnesium citrate, and bisacodyl) were also examined to further investigate the clinical impact of the bowel preparation medications. We note that the use of adjunctive agents is frequently a surrogate for inadequate bowel preparation or difficulty with tolerance.

### Statistical analysis

#### Primary analysis

Characteristics and outcomes of patients in both bowel preparation groups were compared using Mann-Whitney U (19), Poisson, and χ^2^ tests for continuous, count/score, and binary variables, respectively. For continuous and count/score variables, the median and interquartile range (i.e., the 25th and 75th percentiles) and mean and 95% confidence intervals (CIs) were also calculated, respectively.

A propensity score-matching analysis was performed to limit biases in the estimated treatment effect of LV-BP (20). The propensity score (i.e., the probability of receiving LV-BP) of each patient was determined using a multivariate logistic regression model fit on the clinical and hospitalization characteristics presented in Supplemental Table 1 (Supplementary Digital Content 1, http://links.lww.com/CTG/A789) (21). The matched population was created through 1:2 (LV-BP to HV-BP) nearest-neighbor matching, without replacement, using a maximum caliper of 0.1 SD of the estimated propensity score. Propensity score matching was performed using the R package MatchIt (21). If the matching algorithm could not find a 1:2 match within the specified caliper, the algorithm would attempt to match at a lower ratio, and if no match could be found, the unmatched sample was excluded from the analysis.

Using the PSM population, 3 multivariate regression models were developed to assess the association between LV-BP and each of the study outcomes. The multivariate regression models were used to reduce biases in the estimated treatment effect of LV-BP that stem from the nonexact balance of covariates after propensity score matching (20,22). To assess predictors of time to colonoscopy and hospital LOS, multivariate generalized linear models with a Gaussian family and log link were used to account for the right-skewed distribution of the outcome (23,24). The adjusted beta coefficients and corresponding 95% CI of the model were calculated. To assess predictors of adequate bowel preparation, a multivariate logistic regression model was developed and the adjusted odds ratios (ORs) and corresponding 95% CIs were calculated.

To improve model interpretability and reduce overfitting of the final regression models, minimum-maximum scaling of the feature values (25) and feature selection was performed. For additional details, please refer to Supplementary Information (Supplementary Digital Content 1, http://links.lww.com/CTG/A789).

#### Secondary analysis on patient subgroups

Further analysis was performed in 4 subgroups: older inpatients (age ≥ 75 years), inpatients who received opioids before colonoscopy, inpatients with chronic kidney disease, and inpatients whose hospitalization was related to gastrointestinal bleeding. The gastrointestinal bleeding and kidney disease subpopulations were defined as patients with an *International Classification of Disease, 10th revision* or Medicare severity diagnosis-related group related to gastrointestinal bleeding and kidney disease, respectively. For each subpopulation, the propensity scores used for 1:2 matching were recalculated using a multivariate logistic regression model fit on the subpopulation only.

## RESULTS

### Population characteristics and unadjusted outcome comparisons

Among 1,807 inpatients undergoing colonoscopy from January 2018 to January 2021 (full population), 293 (16.2%) and 1,514 (83.2%) patients received LV-BP and HV-BP, respectively. A comparison of the baseline patient characteristics between both groups is provided in Table 1. All 104 characteristics measured in this study are described fully across each group in Supplemental Table 1 (Supplementary Digital Content 1, http://links.lww.com/CTG/A789).

**Table 1.** Summary of baseline characteristics and outcomes of the full study population and 1:2 PSM population

Variables^a^	Full population, n = 1,807			1:2 PSM population, n = 708		
	Low volume, n = 293	High volume, n = 1,514	*P* value	Low volume, n = 249	High volume, n = 459	*P* value
Patient characteristics						
Age, % (N)						
< 45	14.0 (41)	15.4 (233)	0.60	15.7 (39)	15.3 (70)	0.97
45–59	17.1 (50)	18.5 (280)	0.62	19.3 (48)	19.2 (88)	0.95
60–74	39.6 (116)	36.4 (551)	0.33	36.9 (92)	36.8 (169)	0.96
≥ 75	29.4 (86)	29.7 (450)	0.95	28.1 (70)	28.8 (132)	0.93
Sex (male), % (N)	51.5 (151)	55.3 (837)	0.27	51.0 (127)	52.7 (242)	0.72
BMI ≥ 30, % (N)	27.6 (81)	30.3 (458)	0.41	26.9 (67)	27.2 (125)	1.00
Charlson Comorbidity Index, mean (95% CI)	3.5 (3.2–3.7)	3.2 (3.1–3.3)	<0.05	3.2 (3.0–3.5)	3.3 (3.2–3.5)	0.53
Baseline laboratory tests, median (IQR)						
Hemoglobin (g/dL)	9.9 (7.6–11.8)	9.8 (7.5–12.2)	0.41	9.9 (7.6–11.9)	10.4 (7.7–12.4)	0.12
Platelets (10^9^/L)	231.0 (178.0–304.0)	242.0 (179.0–317.0)	0.11	230.0 (177.0–307.0)	240.5 (177.2–309.0)	0.28
Creatinine (mg/dL)	1.0 (0.8–1.4)	1.0 (0.8–1.5)	0.22	1.0 (0.8–1.4)	1.0 (0.8–1.4)	0.49
Glucose (mg/dL)	116.0 (99.0–155.0)	116.0 (100.0–146.0)	0.29	115.0 (99.0–150.0)	116.0 (98.0–147.0)	0.47
Blood urea nitrogen (mg/dL)	17.0 (12.0–27.0)	19.0 (12.0–32.0)	<0.05	17.0 (12.0–28.0)	18.0 (12.0–28.0)	0.38
Potassium (mmol/L)	4.0 (3.7–4.3)	4.1 (3.8–4.5)	<0.01	4.0 (3.7–4.4)	4.1 (3.8–4.4)	0.06
Hospitalization characteristics						
Arrived in ED, % (N)	84.3 (247)	82.0 (1,241)	0.38	83.9 (209)	81.5 (374)	0.48
Was in ICU at any point during hospitalization, % (N)	5.1 (15)	9.4 (143)	<0.05	6.0 (15)	6.3 (29)	0.99
Hospitalization occurred during COVID-19 public health advisories, % (N)	7.8 (23)	8.5 (129)	0.79	9.2 (23)	8.7 (40)	0.92
Discharged to home or self-care, % (N)	61.1 (179)	56.5 (856)	0.17	63.1 (157)	62.7 (288)	1.00
No. of *ICD-10s* associated with stay, mean (95% CI)	21.8 (21.2–22.4)	20.4 (20.2–20.7)	<0.001	21.6 (20.9–22.2)	20.8 (20.4–21.2)	<0.05
Hospitalization related to, % (N)						
Dementia	2.0 (6)	2.8 (43)	0.57	1.6 (4)	2.6 (12)	0.55
GI bleeding	42.7 (125)	56.9 (861)	0.93	47.8 (119)	44.9 (206)	0.51
Cirrhosis	8.2 (24)	8.7 (131)	0.89	9.2 (23)	9.2 (42)	0.92
Diabetes	25.6 (75)	30.6 (463)	0.10	27.3 (68)	28.1 (129)	0.89
Kidney disease	22.2 (65)	27.3 (413)	0.08	23.7 (59)	23.7 (109)	0.94
Medications given before colonoscopy, % (N)						
Antihypertensive	4.1 (12)	9.9 (150)	<0.01	4.8 (12)	4.1 (19)	0.82
Heparin	4.4 (13)	4.5 (68)	0.91	0.4 (1)	0.7 (3)	0.92
Opioid	9.9 (29)	15.5 (234)	<0.05	4.8 (12)	2.4 (11)	0.13
Tricyclic antidepressants	1.4 (4)	1.0 (15)	0.79	11.6 (29)	12.6 (58)	0.79
Warfarin	1.0 (3)	1.6 (24)	0.64	1.2 (3)	1.3 (6)	0.81
Nothing by mouth or liquid diet before colonoscopy, % (N)	100.0 (293)	99.9 (1,512)	<0.001	100.0 (249)	100.0 (459)	1.00
Colonoscopy characteristics						
Colonoscopy performed before noon (12 pm), % (N)	31.7 (93)	38.1 (577)	<0.05	33.7 (84)	34.4 (158)	0.92
Same day EGD and colonoscopy, % (N)	51.2 (150)	40.8 (617)	<0.05	51.8 (129)	47.3 (217)	0.88
ASA class, mean (95% CI)	2.3 (2.2–2.5)	2.1 (2.0–2.2)	<0.05	2.3 (2.1–2.5)	2.3 (2.1–2.4)	0.96
Indication for colonoscopy, % (N)						
Diarrhea	13.5 (36)	9.8 (138)	0.09	12.4 (28)	12.7 (53)	0.97
Hematochezia	26.3 (70)	26.5 (373)	0.99	27.6 (62)	23.9 (100)	0.36
Anemia	21.4 (57)	15.2 (214)	<0.05	20.0 (45)	17.9 (75)	0.59
Abnormal imaging	6.0 (16)	7.6 (107)	0.43	5.8 (13)	8.4 (35)	0.30
Melena	8.3 (22)	8.6 (121)	0.96	8.4 (19)	8.1 (34)	0.99
Inflammatory bowel disease	0.4 (1)	0.1 (1)	0.73	0.4 (1)	0.2 (1)	0.77
Rectal bleeding	4.1 (11)	5.8 (82)	0.34	4.4 (10)	4.5 (19)	0.89
Abdominal pain	1.9 (5)	3.6 (51)	0.21	2.2 (5)	2.9 (12)	0.82
Others	18.4 (49)	22.7 (320)	0.14	19.1 (43)	21.3 (89)	0.58
Outcomes						
Time to colonoscopy (d), median (IQR)	1.1 (0.9–1.9)	1.5 (1.0–2.1)	<0.001	1.2 (0.9–1.9)	1.2 (1.0–2.1)	<0.05
Hospital LOS (d), median (IQR)	5.7 (3.8–9.8)	5.9 (3.7–11.4)	0.17	5.7 (3.8–9.5)	5.8 (3.6–9.8)	0.48
Requirement for additional bowel preparation medication order, % (N)	41.3 (121)	53.4 (809)	0.11	41.8 (104)	52.9 (243)	0.20
Adjunctive laxatives used for bowel preparation,^b^ % (N)	1.4 (4)	10.6 (160)	<0.001	1.6 (4)	9.4 (43)	<0.001
Adequate bowel preparation (Aronchick scale 1 or 2), % (N)	62.1 (146)	58.6 (680)	0.35	60.3 (120)	59.6 (205)	0.94
Aronchick scale rating, % (N)						
1 (Excellent)	31.1 (73)	15.7 (182)	<0.001	29.1 (58)	15.7 (54)	<0.001
2 (Good)	31.1 (73)	42.9 (498)	<0.001	31.2 (62)	43.9 (151)	<0.01
3 (Fair)	11.9 (28)	13.0 (151)	0.73	11.6 (23)	12.2 (42)	0.93
4 (Poor)	14.5 (34)	15.4 (179)	0.79	15.6 (31)	16.0 (55)	1.00
5 (Inadequate)	11.5 (27)	13.0 (151)	0.60	12.6 (25)	12.2 (42)	0.99
Polyp detected, % (N)	31.4 (92)	29.4 (445)	0.54	29.3 (73)	28.3 (130)	0.85
Physician-reported procedure complications, % (N)	0.7 (2)	0.7 (10)	0.73	0.8 (2)	1.7 (8)	0.50
In-hospital mortality, % (N)	1.4 (4)	1.2 (18)	0.97	1.6 (4)	1.3 (6)	0.99
Cecal intubation, % (N)	96.7 (233)	97.0 (1,273)	0.93	96.6 (197)	97.5 (383)	0.72

ASA, American Society of Anesthesiologists; BMI, body mass index; CI, confidence interval; ED, emergency department; EGD, esophagogastroduodenoscopy; GI, gastrointestinal; *ICD-10*, *International Classification of Disease*, 10th revision; ICU, intensive care unit; IQR, interquartile range; LOS, length of stay; PSM, propensity score-matched.

a The number of missing variable values among patients receiving high-volume preparation: baseline laboratory measurements (N = 6), postprocedure laboratory measurements (N = 193), colonoscopy indication (N = 107), time to colonoscopy (N = 22), adequate bowel preparation score (N = 353), and cecal intubation completed (N = 202). The number of missing variable values among patients receiving low-volume preparation: postprocedure laboratory measurements (N = 27), colonoscopy indication (N = 27), time to colonoscopy (N = 5), adequate bowel preparation score (N = 58), and cecal intubation completed (N = 52).

b Adjunctive laxatives defined as enemas, magnesium citrate, and bisacodyl.

While examining unadjusted outcomes within the full population, LV-BP was associated with a significantly shorter time to colonoscopy (median: 1.1 [Interquartile Range (IQR) IQR: 0.9–1.9] vs 1.5 [IQR: 1.0–2.1] days; *P* < 0.001) and with a trend toward shorter hospital LOS (median: 5.7 [IQR: 3.8–9.8] vs 5.9 [IQR: 3.7–11.4] days; *P* = 0.17) compared with HV-BP (Table 1). In addition, although no significant difference was found in the frequency of adequate bowel preparations between LV-BP and HV-BP groups (62.1% vs 58.6%; *P* = 0.35), patients receiving LV-BP had a significantly higher frequency of “Excellent” (Aronchick scale = 1) preparations (31.4% vs 15.4%; *P* < 0.001). No significant differences were seen for in-hospital mortality and physician-reported procedure complication rates between the 2 groups.

Through 1:2 propensity score matching, 249/293 patients (85.0%) who received LV-BP were matched to 459/1,514 patients (30.3%) who received HV-BP. A comparison of baseline characteristics of the PSM populations is summarized in Table 1. A comparison of all examined characteristics between the 2 groups is provided in Supplemental Table 2 (Supplementary Digital Content 1, http://links.lww.com/CTG/A789). Unadjusted outcomes in the propensity-matched population were similar to those in the full population (Table 1).

### Multivariate regression modeling for study outcomes

We next developed a multivariate regression model fit on the PSM population to evaluate the adjusted association of LV-BP with each of the study outcomes. For our primary outcome, we found that LV-BP was significantly associated with a decreased time to colonoscopy (adjusted β: −0.43 [95% CI: −0.56 to −0.30]; *P* < 0.001) (Table 2). Among the covariates included in the model, discharge to home/self-care and gastrointestinal bleeding as the primary reasons for hospitalization were significantly associated with the decreased time to colonoscopy. Initial presentation to the emergency department and opioid use were associated with an increased time to colonoscopy. Among secondary outcomes, we found that LV-BP was associated with a trend toward decreased hospital LOS (adjusted β: −0.09 [95% CI: −0.21 to 0.02]; *P* < 0.12) (Supplemental Table 3, Supplementary Digital Content 1, http://links.lww.com/CTG/A789). Adequate bowel preparation was not associated with LV-BP (adjusted OR: 1.02 [95% CI: 0.71–1.46]; *P* = 0.92) for HV-BP (Supplemental Table 4, Supplementary Digital Content 1, http://links.lww.com/CTG/A789). Overall, our findings demonstrate that LV-BP is associated with a significantly decreased time to colonoscopy without a difference in the frequency of adequate bowel preparation.

**Table 2.** Predictors of time to colonoscopy identified in multivariate regression analysis

Variable	β (95% CI)	*P* value
Received LV-BP	−0.43 (−0.56 to −0.30)	<0.001
Age ≥ 75 yr	0.08 (−0.03 to 0.19)	0.16
Arrived in ED	0.33 (0.18 to 0.47)	<0.001
Discharge to home or self-care	−0.20 (−0.31 to −0.09)	<0.001
Was in the ICU at any point during hospitalization	0.11 (−0.05 to 0.26)	0.20
Hospitalization occurred during COVID-19 public health advisories	0.02 (−0.16 to 0.19)	0.84
Number of *ICD-10s* associated with stay	0.54 (0.22 to 0.86)	<0.001
No complications occurred during hospitalization (based on MS-DRG)	−0.01 (−0.16 to 0.15)	0.92
Hospitalization related to gastrointestinal bleeding	−0.33 (−0.44 to −0.22)	<0.001
Hospitalization related to chronic kidney disease	0.06 (−0.05 to 0.17)	0.31
Opioid use	0.49 (0.38 to 0.60)	<0.001
No. of bowel preparation orders before colonoscopy	1.63 (1.42 to 1.84)	<0.001

The adjusted association of covariates (β coefficients presented) from a multivariate generalized linear model developed using the 1:2 propensity-matched population and variable feature selection (N = 704).

CI, confidence interval; ED, emergency department; *ICD-10*, *International Classification of Disease, 10th revision*; ICU, intensive care unit; LV-BP, low-volume bowel preparation; MS-DRG, Medicare severity diagnosis-related group.

### Secondary analysis on patient subgroups

To perform exploratory investigations as to how LV-BP may affect outcomes in specific patient groups, we performed subgroup analyses for inpatients older than 75 years, receiving opioids before colonoscopy, with chronic kidney disease, and hospitalized for gastrointestinal bleeding. These patient groups were selected because rapid and adequate bowel preparation either is critical for their clinical management (26,27) or represents known risk factors for inadequate bowel preparation or complications (28,29). The matched subpopulation characteristics after 1:2 propensity score matching using propensity scores for LV-BP administration developed on each subpopulation are summarized in Supplementary Tables 5–8 (Supplementary Digital Content 1, http://links.lww.com/CTG/A789). The unadjusted patient outcomes in each of the matched subpopulations are presented in Table 3.

**Table 3.** Patient outcomes among the four 1:2 propensity score-matched subpopulations

Outcomes	Gastrointestinal bleeding			Chronic kidney disease			Received opioids before colonoscopy			Age ≥ 75 yr		
	Low volume, n = 102	High volume, n = 177	*P* value	Low volume, n = 34	High volume, n = 55	*P* value	Low volume, n = 20	High volume, n = 33	*P* value	Low volume, n = 50	High volume, n = 86	*P* value
Time to colonoscopy (d), median (IQR)	1.1 (0.9–1.9)	1.1 (0.9–1.8)	0.31	1.2 (0.9–2.0)	1.4 (1.0–2.0)	0.22	1.8 (1.2–2.1)	2.0 (1.1–3.0)	0.12	1.0 (0.8–1.9)	1.5 (1.0–2.4)	<0.01
Hospital LOS (d), median (IQR)	5.2 (3.7–8.9)	5.6 (3.5–10.4)	0.44	7.5 (5.8–12.6)	7.9 (4.0–14.4)	0.37	5.7 (4.5–10.6)	6.9 (5.5–11.7)	0.09	5.1 (4.3–10.9)	6.6 (3.9–11.7)	0.30
Requirement for additional bowel preparation medication order, % (N)	42.2 (43)	48.0 (85)	0.41	44.1 (15)	49.1 (27)	0.81	85.0 (17)	60.6 (20)	0.12	42.0 (21)	57.0 (49)	0.95
Adjunctive laxatives used for bowel preparation,^a^ % (N)	0.0 (0)	10.7 (19)	<0.01	0.0 (0)	10.9 (6)	0.12	0.0 (0)	18.2 (6)	0.12	0.0 (0)	16.3 (14)	<0.01
Adequate bowel preparation (Aronchick scale 1 or 2), % (N)	61.3 (49)	49.3 (70)	0.17	62.1 (18)	58.1 (25)	0.93	50.0 (8)	46.2 (12)	0.94	58.3 (21)	61.9 (39)	0.89
Aronchick scale rating, % (N)												
1 (Excellent)	37.5 (30)	13.4 (19)	<0.001	31.0 (9)	11.6 (5)	0.08	31.3 (5)	3.8 (1)	<0.05	30.6 (11)	20.6 (13)	0.39
2 (Good)	23.8 (19)	35.9 (51)	0.09	31.0 (9)	46.5 (20)	0.29	18.8 (3)	42.3 (11)	0.22	27.8 (10)	41.3 (26)	0.26
3 (Fair)	12.5 (10)	16.9 (24)	0.50	20.7 (6)	16.3 (7)	0.87	25.0 (4)	11.5 (3)	0.48	13.9 (5)	9.5 (6)	0.74
4 (Poor)	15.0 (12)	22.5 (32)	0.24	10.3 (3)	14.0 (6)	0.93	12.5 (2)	19.2 (5)	0.89	19.4 (7)	17.5 (11)	0.98
5 (Inadequate)	11.3 (9)	11.3 (16)	0.83	6.9 (2)	11.6 (5)	0.80	12.5 (2)	23.1 (6)	0.66	8.3 (3)	11.1 (7)	0.93
Polyp detected, % (N)	31.4 (32)	31.1 (55)	0.93	35.3 (12)	34.5 (19)	0.88	20.0 (4)	36.4 (12)	0.34	34.0 (17)	37.2 (32)	0.85
Physician-reported procedure complications, % (N)	1.0 (1)	0.6 (1)	0.73	0.0 (0)	1.8 (1)	0.81	0.0 (0)	0.0 (0)	1.00	0.0 (0)	0.0 (0)	1.00
In-hospital mortality, % (N)	1.0 (1)	1.1 (2)	0.63	2.9 (1)	1.8 (1)	0.70	5.0 (1)	0.0 (0)	0.80	2.0 (1)	2.3 (2)	0.63
Cecal intubation completed, % (N)	95.1 (77)	98.0 (147)	0.40	96.0 (24)	98.0 (50)	0.81	88.9 (16)	96.3 (26)	0.71	91.7 (33)	98.7 (74)	0.19

IQR, interquartile range; LOS, length of stay.

a Adjunctive laxatives defined as enemas, magnesium citrate, and bisacodyl.

**Figure 1.**
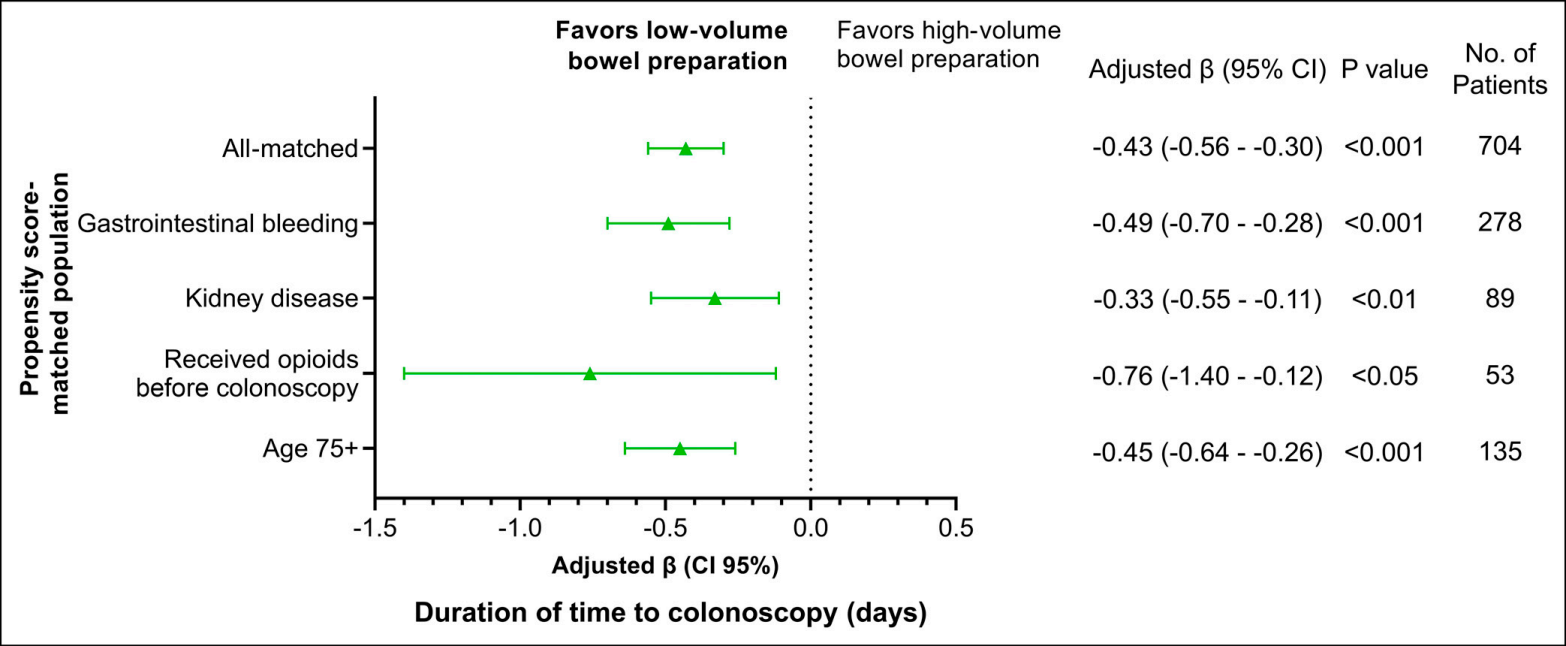
Adjusted association of low-volume bowel preparation with time to colonoscopy in propensity score-matched population and subpopulations. CI, confidence interval.

LV-BP was associated with a significant decrease in time to colonoscopy among all 4 subgroups (Figure 1). The impact of LV-BP on the primary outcome was largest among patients who received opioids before the colonoscopy (adjusted β: −0.76 [95% CI: −1.40 to −0.12]; *P* < 0.05), followed by patients presenting with gastrointestinal bleeding (adjusted β: −0.49 [95% CI: −0.70 to −0.28]; *P* < 0.001), older patients (adjusted β: −0.45 [95% CI: −0.64 to −0.26]; *P* < 0.001), and patients with chronic kidney disease (adjusted β: −0.33 [95% CI: −0.55 to −0.11]; *P* < 0.01).

**Figure 2.**
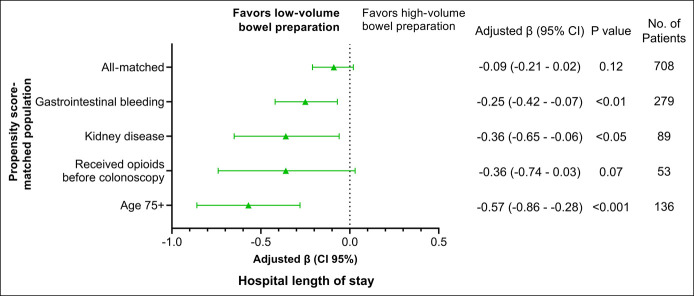
Adjusted association of low-volume bowel preparation with hospital length of stay in propensity score-matched population and subpopulations. CI, confidence interval.

The potential impact of LV-BP on decreasing hospital LOS was observed in this matched subgroup analysis (Figure 2). LV-BP was significantly associated with a decreased LOS among patients hospitalized with gastrointestinal bleeding (adjusted β: −0.25 [95% CI: −0.42 to −0.07]; *P* < 0.01), patients with chronic kidney disease (adjusted β: −0.36 [95% CI: −0.65 to −0.06]; *P* < 0.05), and older patients (adjusted β: −0.57 [95% CI: −0.86 to −0.28]; *P* < 0.001). LV-BP was associated with a trend toward decreased LOS among patients who received opioids before the colonoscopy (adjusted β: −0.36 [95% CI: −0.65 to 0.03]; *P* = 0.07).

**Figure 3.**
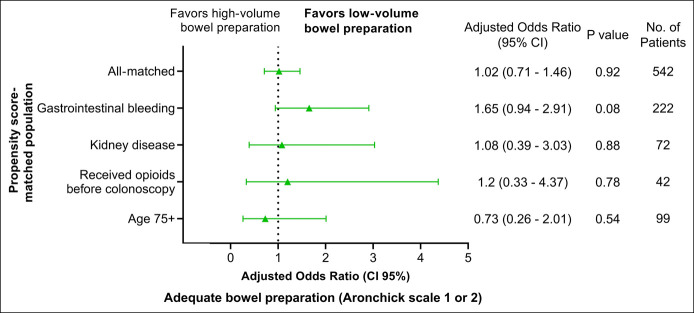
Adjusted association of low-volume bowel preparation with adequate bowel preparation (Aronchick scale 1 or 2) in propensity score-matched population and subpopulations. CI, confidence interval.

Finally, the odds of adequate bowel preparation with LV-BP were not significantly different compared with HV-BP in any of the subpopulations (Figure 3). However, LV-BP was associated with a near-significant trend toward increased odds of adequate preparation among patients hospitalized with gastrointestinal bleeding (adjusted OR: 1.65 [95% CI: 0.94–2.91]; *P* = 0.08).

## DISCUSSION

In this study, we performed a retrospective PSM analysis assessing the impact of LV-BP on hospitalized patients undergoing colonoscopy. We found that LV-BP administration was associated with a significantly decreased time to colonoscopy compared with HV-BP. In addition, we found that LV-BP use was significantly associated with decreased hospital LOS for patients undergoing inpatient colonoscopy for gastrointestinal bleeding, for kidney disease, and for older patients (age ≥ 75 years), as well as strongly associated with decreased LOS for patients receiving opioids, suggesting that the benefits of the reduced preparation completion time may ultimately improve patient flow. Collectively, these results suggest that LV-BP use may reduce delays to inpatient endoscopy, which may potentially lead to improvements in hospital LOS and overall expenditures.

The primary finding in our study was that LV-BP was associated with decreased time to colonoscopy in hospitalized patients compared with HV-BP. Importantly, the reduced time to colonoscopy occurred without reduction in the likelihood of adequate bowel preparation. In fact, patients given LV-BP had a significantly higher proportion of “Excellent” bowel preparation scores compared with those given HV-BP. Although the value of “Excellent” bowel preparation in comparison with that of “Good” for diagnostic yield warrants further investigation and may only lead to limited diagnostic benefits (3, 30), high quality of preparation has been shown to be associated with significantly shorter and less complex procedures (3). Our findings are important given the historical difficulties of developing a sustainable means to improve bowel preparation among hospitalized patients (10–12). Moreover, the importance of time to inpatient endoscopy to quality patient care has recently been underscored by a large retrospective study that identified inpatient endoscopy delay to prolong hospital LOS by a median of 2 days and to independently predict 30-day readmission (31). Therefore, our findings identify LV-BP use as a potential strategy for decreasing endoscopy delays in hospitalized patients undergoing colonoscopy.

We also found that LV-BP may lead to decreased hospital LOS among certain inpatient subpopulations, indicating the potential benefits of LV-BP in reducing hospital costs and improving patient flow. For instance, we found that LV-BP was associated with a reduced hospital LOS among patients hospitalized specifically for gastrointestinal bleeding, a population in which adequate bowel preparation is particularly important to definitively identify and treat a bleeding source (26, 27). Reduced LOS was also found among older populations (age ≥ 75 years). Because older patients often have difficulties drinking high volumes of preparation required for cleansing and commonly suffer from nausea and abdominal pain during preparation (32, 33), the lower volume of LV-BP may be especially beneficial in improving care among this subpopulation. Similarly, we found LV-BP to be beneficial in reducing LOS in patients with chronic kidney disease and those receiving opioids—2 groups that have historically had difficulties with achieving adequate bowel preparation (1, 8). Among the other secondary outcomes we assessed, we notably found that adjunctive laxative medications (i.e., enemas, magnesium citrate, and bisacodyl) were used significantly more frequently in the HV-BP group, suggesting that LV-BP may intrinsically lead to superior bowel preparation (i.e., without requiring adjunctive laxatives). LV-BP use was also associated with a lower rate of requiring additional preparation compared with HV-BP use, further emphasizing the effectiveness and improvements in tolerance of LV-BP.

Among our overall population, we found that LV-BP was associated with a nonsignificant trend toward reduced LOS. This may be because relatively healthy patients with fewer comorbidities who are able to tolerate high-volume preparations would not be expected to see the same benefits of using lower volume preparations. In addition, hospital LOS is an outcome that depends on a complex interplay between multiple patient and hospital-related factors (34), which extend beyond bowel preparation tolerance, duration, and quality. As such, we may also have been underpowered to detect a significant difference in hospital LOS in the overall population.

Importantly, we noted no increased adverse events in the LV-BP group compared with the HV-BP group. Low-volume preparations are hyperosmotic agents that carry theoretical risks of electrolyte disturbances and hypovolemia, particularly in patients with cardiac, renal, or hepatic dysfunction. This is particularly salient in the hospitalized population because there is a greater prevalence of such comorbidities, compared with outpatient populations. Our results suggest that with careful monitoring of laboratory results and administration of fluid as necessary, LV-BP use is likely safe for use in hospitalized patients.

Our study had several limitations. First, we were limited by the retrospective nature of our study, which may lead to bias. However, we were able to use a propensity score-matched analysis that allowed us to account for many variables that may be important to our outcomes of interest. Moreover, each multivariate regression model controlled for patient and hospitalization characteristics identified through a feature selection algorithm, further reducing possible biases stemming from imperfect matches after propensity-matched analysis. In addition, although we could not assess the role of endoscopy scheduling and anesthesia availability to potential delays to colonoscopy, the fact that the endoscopy suite at the medical center examined in this study is open 7 days a week with a dedicated procedure room with anesthesia availability should mitigate such contributions. Second, we were unable to gather data explicitly describing tolerability of preparation. However, the decreased time to colonoscopy and similar rates of adverse events between the 2 groups strongly suggest that LV-BP is at least as well tolerated than standard HV-BP. Third, bowel preparation quality was documented according to the Aronchick scale, which has large interobserver variability compared with other scales including the Boston Bowel Preparation Scale (35). Fourth, detailed information on patient behaviors and adherence to preprocedural diet and medication orders, such as exact time stamps related to when patients last ingested bowel preparation medications before the colonoscopy, were unavailable and could conceivably have impact on our investigated outcomes. However, those delays in inpatient endoscopy due to compliance with NPO status or late drinking of preparations would likely affect both groups equally and would not appreciably affect the overall findings. Fifth, as we only compared Suprep with GoLYTELY, we cannot make a definitive conclusion regarding whether other LV-BPs (i.e., Moviprep, Plenvu, and Clenpiq) would perform in a comparable manner.

Finally, our study was a single-center analysis, which limits generalizability. However, to the best of our knowledge, our study is the largest to date specifically exploring the impact of a LV-BP formulation on hospitalized patients undergoing colonoscopy. Moreover, our patient population was diverse, and thus, our study should be representative of most tertiary and quaternary care hospitals.

Our study demonstrates that LV-BP use among inpatient populations can substantially reduce the time from bowel preparation to colonoscopy as well as related avoidable hospital bed days and potentially excess healthcare costs. Moreover, it builds on emerging data that suggest that LV-BPs may lead to similar degrees of bowel preparation quality compared with traditional HV-BPs in the hospitalized setting. Cost-effectiveness analyses and randomized controlled trials comparing the efficacy and tolerability of LV-BPs vs HV-BPs in hospitalized patients are needed to provide support in determining whether the use of LV-BPs should be widely adopted in inpatients requiring bowel preparation for colonoscopy.

## CONFLICTS OF INTEREST

**Guarantor of the article:** James M. Richter, MD.

**Specific author contributions:** Study concept and design: C.L.F.S., D.K.L., A.C.Z., R.L., and J.M.R. Acquisition of data: C.L.F.S. and J.H. Analysis and interpretation of data: C.L.F.S., D.K.L., M.A.B., P.C., B.D., S.E., A.M., K.S., B.J.Y., P.D., R.L., and J.M.R. Manuscript writing: C.L.F.S. and D.K.L. All authors approved the final draft submitted.

**Financial support:** C.L.F.S. is a recipient of a Canadian Institutes of Health Research Fellowship.

**Potential competing interests:** J.M.R. consults with Iterative Scopes (Cambridge, MA) and WorldCare International (Boston, MA). A.M. discloses a financial relationship with Amazon Pharmacy through a family member. All other authors have no disclosures.

**Study Highlights**

**WHAT IS KNOWN**

✓ Delays in inpatient colonoscopy are commonly caused by inadequate bowel preparation.
✓ Few strategies exist to reduce the risk of inadequate bowel preparation in hospitalized patients.

**WHAT IS NEW HERE**

✓ Low-volume bowel preparation (LV-BP) use in hospitalized patients is associated with decreased time to colonoscopy.
✓ LV-BP is associated with a similar frequency of adequate bowel preparation compared with traditional high-volume bowel preparation.
✓ LV-BP is associated with decreased hospital length of stay in older patients, patients with chronic kidney disease, and patients presenting with gastrointestinal bleeding.

## Supplementary Material
