# Supplementary material for: Low-Volume Bowel Preparation Is Associated With Reduced Time to Colonoscopy in Hospitalized Patients: A Propensity-Matched Analysis
Source: Clin Transl Gastroenterol. 2022 Mar 28;13(7):e00482. doi: 10.14309/ctg.0000000000000482 (PMC10476773; doi:10.14309/ctg.0000000000000482)
Supplement: Supplementary file 1 [file ct9-13-e00482-s001.docx]

**Supplementary Information**

**Supplementary Methods**

*Interpretation of the generalized linear model’s coefficients*

A generalized linear model ([1](#_ENREF_1)) (GLM) with a gaussian distribution and log-link can be written as:

$g\left( \mu_{i} \right)=\eta_{i}= {\alpha+\beta}_{i1}X_{i1}+ \beta_{i2}X_{i2}+\ldots+ \beta_{ik}X_{ik}$ $(1)$

Where the link function $g\left( \cdot\right)$ is a logistic function, and the dependent variable $\mu$ follows a gaussian distribution. Accordingly, equation (1) can be re-written as:

$$log\left( \mu_{i} \right)={\alpha+\beta}_{i1}X_{i1}+ \beta_{i2}X_{i2}+\ldots+ \beta_{ik}X_{ik}$$

$\mu_{i}=e^{\alpha+{\beta_{i1}X}_{i1}+ \beta_{i2}X_{i2}+\ldots+ \beta_{ik}X_{ik}}$ $(2)$

Based on equation (2), an estimation of the dependent variable $\hat{\mu}$ can be made as follows:

$$\hat{\mu}_{i}=e^{\alpha+{\beta_{i1}X}_{i1}+ \beta_{i2}X_{i2}+\ldots+\beta_{ij}X_{ij}+\ldots+ \beta_{ik}X_{ik}}$$

For a given change, $N$, in any independent variable $X_{ij}$, we see that:

$$\hat{\mu}_{i}^{'}=e^{\alpha+{\beta_{i1}X}_{i1}+ \beta_{i2}X_{i2}+\ldots+\beta_{ij}{(X}_{ij}+N)+\ldots+ \beta_{ik}X_{ik}}$$

$$\hat{\mu}_{i}^{'}=e^{\alpha+{\beta_{i1}X}_{i1}+ \beta_{i2}X_{i2}+\ldots+\beta_{ij}X_{ij}+\ldots+ \beta_{ik}X_{ik}}\cdot e^{\beta_{ij}(N)}$$

$\hat{\mu}_{i}^{'}=\hat{\mu}_{i}\cdot e^{\beta_{ij}(N)}$ $(3)$

Therefore, the exponentiated coefficients values from the GLM can be interpreted to have a multiplicative effect on the independent variable.

*Feature Selection*

Feature selection for each of the models was conducted through a 10-fold cross-validation (CV) approach, where the number of times each feature was selected by the regression model after L1 regularization in each of the 10 folds was calculated. Features selected 7 or more times out of the 10 CV-folds were included in the final models. Additionally, based on a priori knowledge from field experts, a subset of patient and hospitalization characteristics (i.e., discharge disposition, was in an ICU during hospitalization, if the MS-DRG associated with the stay indicated no complications occurred, and if the hospitalization occurred during Boston’s COVID-19 public health advisories) were controlled for in the final models, regardless of the number of times they were selected over the 10-folds. To estimate the adjusted association of low volume preparations with the study outcomes, the final models were refit on across all inpatients, while adjusting for the selected features and the additional field expert selected characteristics. Patients with missing study outcomes (dependent variable) values were excluded from model fitting. Missing features (independent variables) values were imputed through a k-nearest neighbors approach based on all the patient’s non-missing feature values, using the Python package fancyimpute ([2](#_ENREF_2)).

**Supplemental Table 1. All measured baseline characteristics and outcomes of the full study population.**

^*^The number of missing variable values among patients receiving high volume preparation: Baseline lab measurements (N=6), Post procedure lab measurements (N=193); Colonoscopy Indication (N=107); Adequate bowel preparation score (N=353); Cecal intubation completed (N=202). The number of missing variable values among patients receiving low volume preparation: Post procedure lab measurements (N=27); Colonoscopy Indication (N=27); Adequate bowel preparation score (N=58); Cecal intubation completed (N=52)

| Variable | Low volume, n = 293 | High volume, n = 1514 | P-value | Standardized mean differences |
| --- | --- | --- | --- | --- |
| Patient Characteristics | | | | |
| 44 and below, % (N) | 14.0% (41) | 15.4% (233) | 0.60 | <0.001 |
| 45-59, % (N) | 17.1% (50) | 18.5% (280) | 0.62 | <0.001 |
| 60-74, % (N) | 39.6% (116) | 36.4% (551) | 0.33 | <0.1 |
| 75 and above, % (N) | 29.4% (86) | 29.7% (450) | 0.95 | <0.001 |
| Sex (Male), % (N) | 51.5% (151) | 55.3% (837) | 0.27 | <0.001 |
| BMI above 30, % (N) | 27.6% (81) | 30.3% (458) | 0.41 | <0.001 |
| Charlson Comorbidity Index, mean (95% CI) | 3.5 (3.2 - 3.7) | 3.2 (3.1 - 3.3) | <0.05 | <0.1 |
| AIDS/HIV score, % (N) | 0.3% (1) | 0.5% (7) | 0.78 | <0.001 |
| Cancer score, % (N) | 20.5% (60) | 19.2% (291) | 0.66 | <0.1 |
| Cerebrovascular disease score, % (N) | 7.5% (22) | 6.0% (91) | 0.35 | <0.1 |
| Chronic pulmonary disease score, % (N) | 27.6% (81) | 25.0% (378) | 0.41 | <0.1 |
| Congestive heart failure score, % (N) | 28.7% (84) | 27.2% (412) | 0.66 | <0.1 |
| Dementia score, % (N) | 1.7% (5) | 2.9% (44) | 0.26 | <0.001 |
| Diabetes score, % (N) | 33.1% (97) | 29.7% (450) | 0.34 | <0.1 |
| Hemiplegia/paraplegia score, % (N) | 0.7% (2) | 1.7% (25) | 0.23 | <0.001 |
| Liver disease score, % (N) | 18.8% (55) | 15.3% (232) | 0.18 | <0.1 |
| Myocardial infarction score, % (N) | 13.0% (38) | 12.9% (195) | 0.97 | <0.01 |
| Peptic ulcer disease score, % (N) | 12.6% (37) | 13.0% (197) | 0.87 | <0.001 |
| Peripheral vascular disease score, % (N) | 16.0% (47) | 15.0% (227) | 0.67 | <0.1 |
| Renal disease score, % (N) | 24.6% (72) | 28.2% (427) | 0.28 | <0.001 |
| Rheumatic disease score, % (N) | 7.8% (23) | 4.2% (64) | <0.05 | 0.15 |
| Baseline: Hemoglobin (g/dL), median (IQR) | 9.9 (7.6 - 11.8) | 9.8 (7.5 - 12.2) | 0.41 | <0.001 |
| Baseline: Mean corpuscular volume (fL), median (IQR) | 89.3 (84.1 - 93.6) | 89.3 (83.6 - 94.4) | 0.35 | <0.001 |
| Baseline: Platelets (10^9/L), median (IQR) | 231.0 (178.0 - 304.0) | 242.0 (179.0 - 317.0) | 0.11 | <0.001 |
| Baseline: Red cell distribution width (%), median (IQR) | 15.5 (13.5 - 17.6) | 15.3 (13.5 - 17.5) | 0.29 | <0.1 |
| Baseline: Creatinine (mg/dL), median (IQR) | 1.0 (0.8 - 1.4) | 1.0 (0.8 - 1.5) | 0.22 | <0.001 |
| Baseline: Glucose (mg/dL), median (IQR) | 116.0 (99.0 - 155.0) | 116.0 (100.0 - 146.0) | 0.29 | <0.1 |
| Baseline: White blood cell count (10^9/L), median (IQR) | 7.9 (5.8 - 10.9) | 8.5 (6.4 - 11.3) | <0.05 | <0.001 |
| Baseline: Blood urea nitrogen (mg/dL), median (IQR) | 17.0 (12.0 - 27.0) | 19.0 (12.0 - 32.0) | <0.05 | <0.001 |
| Baseline: Potassium (mmol/L), median (IQR) | 4.0 (3.7 - 4.3) | 4.1 (3.8 - 4.5) | <0.01 | <0.001 |
| Baseline: Sodium (mmol/L), median (IQR) | 138.0 (135.0 - 140.0) | 138.0 (136.0 - 140.0) | 0.08 | <0.001 |
| Baseline: Chloride (mmol/L), median (IQR) | 101.0 (98.0 - 104.0) | 101.0 (97.0 - 104.0) | <0.05 | 0.13 |
| Baseline: Diastolic blood pressure (mmHg), median (IQR) | 70.0 (62.0 - 78.0) | 68.0 (60.0 - 77.0) | 0.14 | <0.1 |
| Baseline: Systolic blood pressure (mmHg), median (IQR) | 134.0 (118.0 - 151.0) | 130.0 (114.0 - 147.8) | <0.05 | <0.1 |
| Baseline: Heart rate (bpm), median (IQR) | 85.0 (74.0 - 98.0) | 83.0 (72.0 - 95.0) | <0.05 | <0.1 |
| Relative difference pre-colonoscopy: Hemoglobin (g/dL), median (IQR) | 0.2 (-0.9 - 1.3) | 0.2 (-1.0 - 1.3) | 0.38 | <0.001 |
| Relative difference pre-colonoscopy: Mean corpuscular volume (fL), median (IQR) | -0.4 (-1.8 - 1.1) | -0.4 (-1.9 - 1.0) | 0.45 | <0.01 |
| Relative difference pre-colonoscopy: Platelets (10^9/L), median (IQR) | 18.0 (-7.0 - 52.0) | 20.0 (-4.0 - 51.0) | 0.27 | <0.01 |
| Relative difference pre-colonoscopy: Red cell distribution width (%), median (IQR) | -0.1 (-0.5 - 0.2) | -0.1 (-0.7 - 0.2) | 0.38 | <0.1 |
| Relative difference pre-colonoscopy: Creatinine (mg/dL), median (IQR) | 0.1 (0.0 - 0.3) | 0.1 (0.0 - 0.3) | <0.01 | <0.001 |
| Relative difference pre-colonoscopy: Glucose (mg/dL), median (IQR) | 17.0 (0.0 - 42.0) | 16.0 (0.0 - 40.0) | 0.39 | <0.001 |
| Relative difference pre-colonoscopy: White blood cell count (10^9/L), median (IQR) | 0.9 (-0.2 - 2.6) | 1.4 (0.0 - 3.2) | <0.01 | <0.001 |
| Relative difference pre-colonoscopy: Blood urea nitrogen (mg/dL), median (IQR) | 5.0 (1.0 - 10.0) | 6.0 (2.0 - 13.0) | <0.05 | <0.001 |
| Relative difference pre-colonoscopy: Potassium (mmol/L), median (IQR) | 0.2 (-0.2 - 0.5) | 0.2 (-0.1 - 0.6) | 0.12 | <0.001 |
| Relative difference pre-colonoscopy: Sodium (mmol/L), median (IQR) | -2.0 (-5.0 - 0.0) | -1.0 (-4.0 - 1.0) | <0.001 | <0.001 |
| Relative difference pre-colonoscopy: Chloride (mmol/L), median (IQR) | -3.0 (-6.0 - 0.0) | -3.0 (-6.0 - 0.0) | 0.49 | <0.1 |
| Relative difference pre-colonoscopy: Diastolic blood pressure (mmHg), median (IQR) | 4.0 (-5.0 - 12.0) | 2.0 (-6.0 - 11.0) | 0.11 | <0.1 |
| Relative difference pre-colonoscopy: Systolic blood pressure (mmHg), median (IQR) | 7.0 (-7.0 - 22.0) | 5.0 (-11.0 - 21.0) | 0.08 | <0.1 |
| Relative difference pre-colonoscopy: Heart rate (bpm), median (IQR) | 7.0 (-2.0 - 18.0) | 5.0 (-4.0 - 17.0) | <0.05 | <0.1 |
| Relative difference post-colonoscopy: Hemoglobin (g/dL), median (IQR) | 0.3 (-0.8 - 1.2) | 0.3 (-1.1 - 1.6) | 0.39 | <0.001 |
| Relative difference post-colonoscopy: Mean corpuscular volume (fL), median (IQR) | -0.9 (-2.7 - 0.5) | -0.8 (-2.4 - 0.9) | 0.19 | <0.001 |
| Relative difference post-colonoscopy: Platelets (10^9/L), median (IQR) | 21.0 (-3.5 - 52.0) | 22.0 (-9.0 - 56.0) | 0.48 | <0.1 |
| Relative difference post-colonoscopy: Red cell distribution width (%), median (IQR) | -0.2 (-0.7 - 0.2) | -0.2 (-0.8 - 0.2) | 0.43 | <0.1 |
| Relative difference post-colonoscopy: Creatinine (mg/dL), median (IQR) | 0.1 (0.0 - 0.3) | 0.1 (-0.0 - 0.3) | 0.17 | <0.001 |
| Relative difference post-colonoscopy: Glucose (mg/dL), median (IQR) | 7.5 (-13.0 - 33.8) | 10.0 (-11.0 - 36.0) | 0.20 | <0.001 |
| Relative difference post-colonoscopy: White blood cell count (10^9/L), median (IQR) | 0.9 (-0.6 - 2.3) | 1.3 (-0.3 - 3.3) | <0.01 | <0.001 |
| Relative difference post-colonoscopy: Blood urea nitrogen (mg/dL), median (IQR) | 5.0 (1.0 - 12.0) | 7.0 (2.0 - 14.0) | <0.05 | <0.001 |
| Relative difference post-colonoscopy: Potassium (mmol/L), median (IQR) | 0.1 (-0.2 - 0.5) | 0.2 (-0.2 - 0.6) | 0.09 | <0.001 |
| Relative difference post-colonoscopy: Sodium (mmol/L), median (IQR) | -2.0 (-4.0 - 1.0) | -1.0 (-4.0 - 1.0) | 0.08 | <0.001 |
| Relative difference post-colonoscopy: Chloride (mmol/L), median (IQR) | -3.0 (-6.0 - 0.0) | -3.0 (-6.0 - 0.0) | 0.22 | <0.1 |
| Relative difference post-colonoscopy: Diastolic blood pressure (mmHg), median (IQR) | 4.0 (-5.0 - 12.0) | 2.0 (-6.0 - 11.2) | 0.11 | <0.1 |
| Relative difference post-colonoscopy: Systolic blood pressure (mmHg), median (IQR) | 7.0 (-7.0 - 22.0) | 5.0 (-11.0 - 21.0) | 0.08 | <0.1 |
| Relative difference post-colonoscopy: Heart rate (bpm), median (IQR) | 7.0 (-2.0 - 18.0) | 5.0 (-4.0 - 17.0) | <0.05 | <0.1 |
| Hospitalization Characteristics | | | | |
| Admit Source: Self Referral, % (N) | 56.7% (166) | 52.2% (790) | 0.18 | <0.1 |
| Arrived in ED, % (N) | 84.3% (247) | 82.0% (1,241) | 0.38 | <0.1 |
| Was in an ICU at any point during hospitalization, % (N) | 5.1% (15) | 9.4% (143) | <0.05 | <0.001 |
| Hospitalization occurred during COVID-19 public health advisories, % (N) | 7.8% (23) | 8.5% (129) | 0.79 | <0.001 |
| Discharge Disposition: Home or Self-care, % (N) | 61.1% (179) | 56.5% (856) | 0.17 | <0.1 |
| Number of ICD10s associated with stay, mean (95% CI) | 21.8 (21.2 - 22.4) | 20.4 (20.2 - 20.7) | <0.001 | 0.15 |
| No complications occurred during hospitalization (based on MS-DRG), % (N) | 15.7% (46) | 23.0% (348) | <0.01 | <0.001 |
| Hospitalization related to: Dementia, % (N) | 2.0% (6) | 2.8% (43) | 0.57 | <0.001 |
| Hospitalization related to: GI Bleed, % (N) | 42.7% (125) | 56.9% (861) | 0.93 | <0.001 |
| Hospitalization related to: Cirrhosis, % (N) | 8.2% (24) | 8.7% (131) | 0.89 | <0.001 |
| Hospitalization related to: Diabetes, % (N) | 25.6% (75) | 30.6% (463) | 0.10 | <0.001 |
| Hospitalization related to: Kidney Disease, % (N) | 22.2% (65) | 27.3% (413) | 0.08 | <0.001 |
| Medication ordered before colonoscopy: Antihypertensive, % (N) | 4.1% (12) | 9.9% (150) | <0.01 | <0.001 |
| Medication ordered before colonoscopy: Heparin, % (N) | 4.4% (13) | 4.5% (68) | 0.91 | <0.001 |
| Medication ordered before colonoscopy: Opioid, % (N) | 9.9% (29) | 15.5% (234) | <0.05 | <0.001 |
| Medication ordered before colonoscopy: Tricyclic antidepressant, % (N) | 0.3% (1) | 0.6% (9) | 0.92 | <0.001 |
| Medication ordered before colonoscopy: Warfarin, % (N) | 1.0% (3) | 1.6% (24) | 0.64 | <0.001 |
| Medication ordered after colonoscopy: Antihypertensive, % (N) | 8.5% (25) | 16.3% (247) | <0.001 | <0.001 |
| Medication ordered after colonoscopy: Heparin, % (N) | 6.5% (19) | 7.5% (113) | 0.64 | <0.001 |
| Medication ordered after colonoscopy: Opioid, % (N) | 26.3% (77) | 40.4% (611) | <0.001 | <0.001 |
| Medication ordered after colonoscopy: Tricyclic antidepressant, % (N) | 1.4% (4) | 1.0% (15) | 0.79 | <0.1 |
| Medication ordered after colonoscopy: Warfarin, % (N) | 7.5% (22) | 6.2% (94) | 0.48 | <0.1 |
| Nothing by mouth or liquid diet before colonoscopy, % (N) | 100.0% (293) | 99.9% (1,512) | <0.001 | <0.1 |
| Number of all blood transfusions given during hospitalization, mean (95% CI) | 3.8 (3.6 - 4.1) | 5.1 (4.9 - 5.2) | <0.001 | <0.001 |
| Number of blood transfusions given before procedure, mean (95% CI) | 0.5 (0.4 - 0.5) | 0.9 (0.9 - 1.0) | <0.001 | <0.001 |
| Number of blood transfusions given after procedure, mean (95% CI) | 1.5 (1.4 - 1.6) | 2.1 (2.0 - 2.2) | <0.001 | <0.001 |
| Number of surgical procedures during hospitalization, mean (95% CI) | 2.5 (2.3 - 2.7) | 2.5 (2.5 - 2.6) | 0.54 | <0.001 |
| Number of surgical procedures between bowel preparation order and colonoscopy, mean (95% CI) | 0.0 (-0.003 - 0.01) | 0.1 (0.04 - 0.06) | <0.01 | <0.001 |
| Number of surgical procedures after colonoscopy, mean (95% CI) | 0.3 (0.2 - 0.3) | 0.4 (0.3 - 0.3) | <0.05 | <0.001 |
| Colonoscopy Characteristics | | | | |
| Colonoscopy performed before noon (12 PM), % (N) | 31.7% (93) | 38.1% (577) | <0.05 | <0.001 |
| Same-day double exam: Colonoscopy and esophagogastroduodenoscopy, % (N) | 51.2% (150) | 40.8% (617) | <0.05 | 0.21 |
| Same-day double exam: Colonoscopy performed with any another endoscopy procedure, % (N) | 3.8% (11) | 7.0% (106) | 0.05 | <0.001 |
| American Society Of Anesthesiologists Rating, mean (95% CI) | 2.3 (2.2 - 2.5) | 2.1 (2.0 - 2.2) | <0.05 | 0.17 |
| Indication: Diarrhea, % (N) | 13.5% (36) | 9.8% (138) | 0.09 | 0.12 |
| Indication: Hematochezia, % (N) | 26.3% (70) | 26.5% (373) | 0.99 | <0.001 |
| Indication: Anemia, % (N) | 21.4% (57) | 15.2% (214) | <0.05 | 0.16 |
| Indication: Abnormal imaging, % (N) | 6.0% (16) | 7.6% (107) | 0.43 | <0.001 |
| Indication: Melena, % (N) | 8.3% (22) | 8.6% (121) | 0.96 | <0.001 |
| Indication: Inflammatory bowel disease, % (N) | 0.4% (1) | 0.1% (1) | 0.73 | <0.1 |
| Indication: Rectal bleeding, % (N) | 4.1% (11) | 5.8% (82) | 0.34 | <0.001 |
| Indication: Abdominal pain, % (N) | 1.9% (5) | 3.6% (51) | 0.21 | <0.001 |
| Indication: Other, % (N) | 18.4% (49) | 22.7% (320) | 0.14 | <0.001 |

**Supplemental Table 2. All measured baseline characteristics and outcomes of the 1:2 propensity score matched population.**

^*^The number of missing variable values among patients receiving high volume preparation: Baseline lab measurements (N=3), Post procedure lab measurements (N=63); Colonoscopy Indication (N=26); Adequate bowel preparation score (N=116); Cecal intubation completed (N=67). The number of missing variable values among patients receiving low volume preparation: Post procedure lab measurements (N=26); Colonoscopy Indication (N=24); Adequate bowel preparation score (N=50); Cecal intubation completed (N=45)

| Variable | Low volume, n = 249 | High volume, n = 459 | P-value | Standardized mean differences |
| --- | --- | --- | --- | --- |
| Patient Characteristics | | | | |
| 44 and below, % (N) | 15.7% (39) | 15.5% (71) | 0.97 | <0.1 |
| 45-59, % (N) | 19.3% (48) | 19.4% (89) | 0.95 | <0.001 |
| 60-74, % (N) | 36.5% (91) | 36.8% (169) | 0.99 | <0.001 |
| 75 and above, % (N) | 28.5% (71) | 28.3% (130) | 0.97 | <0.01 |
| Sex (Male), % (N) | 51.0% (127) | 53.6% (246) | 0.56 | <0.001 |
| BMI above 30, % (N) | 26.9% (67) | 27.2% (125) | 1.00 | <0.001 |
| Charlson Comorbidity Index, mean (95% CI) | 3.2 (3.0 - 3.5) | 3.3 (3.2 - 3.5) | 0.58 | <0.001 |
| AIDS/HIV score, % (N) | 0.4% (1) | 0.9% (4) | 0.49 | <0.001 |
| Cancer score, % (N) | 19.7% (49) | 20.3% (93) | 0.87 | <0.001 |
| Cerebrovascular disease score, % (N) | 6.8% (17) | 6.5% (30) | 0.89 | <0.1 |
| Chronic pulmonary disease score, % (N) | 26.9% (67) | 30.1% (138) | 0.46 | <0.001 |
| Congestive heart failure score, % (N) | 24.9% (62) | 24.6% (113) | 0.94 | <0.01 |
| Dementia score, % (N) | 1.6% (4) | 2.6% (12) | 0.40 | <0.001 |
| Diabetes score, % (N) | 28.5% (71) | 28.8% (132) | 0.95 | <0.001 |
| Hemiplegia/paraplegia score, % (N) | 0.8% (2) | 0.9% (4) | 0.93 | <0.001 |
| Liver disease score, % (N) | 18.9% (47) | 18.7% (86) | 0.97 | <0.01 |
| Myocardial infarction score, % (N) | 11.2% (28) | 12.2% (56) | 0.73 | <0.001 |
| Peptic ulcer disease score, % (N) | 13.3% (33) | 11.1% (51) | 0.43 | <0.1 |
| Peripheral vascular disease score, % (N) | 16.1% (40) | 18.5% (85) | 0.46 | <0.001 |
| Renal disease score, % (N) | 24.1% (60) | 24.6% (113) | 0.89 | <0.001 |
| Rheumatic disease score, % (N) | 7.6% (19) | 7.2% (33) | 0.84 | <0.1 |
| Baseline: Hemoglobin (g/dL), median (IQR) | 9.8 (7.5 - 11.9) | 10.4 (7.7 - 12.4) | 0.10 | <0.001 |
| Baseline: Mean corpuscular volume (fL), median (IQR) | 89.3 (84.0 - 93.5) | 89.3 (84.1 - 94.4) | 0.26 | <0.001 |
| Baseline: Platelets (10^9/L), median (IQR) | 231.0 (178.0 - 309.0) | 240.0 (177.2 - 308.0) | 0.35 | <0.1 |
| Baseline: Red cell distribution width (%), median (IQR) | 15.4 (13.4 - 17.6) | 15.2 (13.5 - 17.4) | 0.26 | <0.1 |
| Baseline: Creatinine (mg/dL), median (IQR) | 1.0 (0.8 - 1.4) | 1.0 (0.8 - 1.4) | 0.50 | <0.001 |
| Baseline: Glucose (mg/dL), median (IQR) | 116.0 (99.0 - 150.0) | 115.0 (99.0 - 147.0) | 0.49 | <0.001 |
| Baseline: White blood cell count (10^9/L), median (IQR) | 8.2 (5.8 - 11.1) | 8.4 (6.1 - 11.1) | 0.28 | <0.1 |
| Baseline: Blood urea nitrogen (mg/dL), median (IQR) | 17.0 (12.0 - 28.0) | 18.0 (12.0 - 28.0) | 0.41 | <0.1 |
| Baseline: Potassium (mmol/L), median (IQR) | 4.0 (3.7 - 4.4) | 4.1 (3.8 - 4.4) | 0.08 | <0.001 |
| Baseline: Sodium (mmol/L), median (IQR) | 138.0 (135.0 - 140.0) | 138.0 (135.0 - 140.0) | 0.36 | <0.001 |
| Baseline: Chloride (mmol/L), median (IQR) | 101.0 (97.0 - 104.0) | 101.0 (97.0 - 104.0) | 0.36 | <0.1 |
| Baseline: Diastolic blood pressure (mmHg), median (IQR) | 70.0 (63.0 - 78.0) | 69.0 (60.0 - 77.0) | 0.21 | <0.1 |
| Baseline: Systolic blood pressure (mmHg), median (IQR) | 133.0 (118.0 - 150.0) | 132.0 (115.0 - 148.5) | 0.30 | <0.1 |
| Baseline: Heart rate (bpm), median (IQR) | 85.0 (74.0 - 98.0) | 84.0 (74.0 - 97.0) | 0.41 | <0.001 |
| Relative difference pre-colonoscopy: Hemoglobin (g/dL), median (IQR) | 0.2 (-0.9 - 1.4) | 0.2 (-1.0 - 1.3) | 0.39 | <0.001 |
| Relative difference pre-colonoscopy: Mean corpuscular volume (fL), median (IQR) | -0.4 (-1.9 - 0.9) | -0.2 (-1.8 - 1.1) | 0.14 | <0.001 |
| Relative difference pre-colonoscopy: Platelets (10^9/L), median (IQR) | 17.0 (-9.0 - 52.0) | 19.0 (-3.0 - 49.0) | 0.31 | <0.1 |
| Relative difference pre-colonoscopy: Red cell distribution width (%), median (IQR) | -0.1 (-0.5 - 0.2) | -0.1 (-0.5 - 0.2) | 0.48 | <0.001 |
| Relative difference pre-colonoscopy: Creatinine (mg/dL), median (IQR) | 0.1 (0.0 - 0.3) | 0.1 (0.0 - 0.3) | 0.20 | <0.001 |
| Relative difference pre-colonoscopy: Glucose (mg/dL), median (IQR) | 17.0 (0.0 - 42.0) | 15.0 (-1.0 - 41.0) | 0.31 | <0.001 |
| Relative difference pre-colonoscopy: White blood cell count (10^9/L), median (IQR) | 0.9 (-0.2 - 2.7) | 1.3 (0.0 - 3.1) | <0.05 | <0.001 |
| Relative difference pre-colonoscopy: Blood urea nitrogen (mg/dL), median (IQR) | 5.0 (1.0 - 10.0) | 6.0 (1.0 - 11.0) | 0.34 | <0.001 |
| Relative difference pre-colonoscopy: Potassium (mmol/L), median (IQR) | 0.2 (-0.2 - 0.5) | 0.2 (-0.1 - 0.5) | 0.32 | <0.001 |
| Relative difference pre-colonoscopy: Sodium (mmol/L), median (IQR) | -2.0 (-4.0 - 0.0) | -2.0 (-5.0 - 0.0) | 0.46 | <0.001 |
| Relative difference pre-colonoscopy: Chloride (mmol/L), median (IQR) | -3.0 (-6.0 - 0.0) | -3.0 (-6.0 - 0.0) | 0.39 | <0.1 |
| Relative difference pre-colonoscopy: Diastolic blood pressure (mmHg), median (IQR) | 5.0 (-4.0 - 12.0) | 4.0 (-5.0 - 13.0) | 0.49 | <0.01 |
| Relative difference pre-colonoscopy: Systolic blood pressure (mmHg), median (IQR) | 7.0 (-7.0 - 21.0) | 7.0 (-8.0 - 23.0) | 0.35 | <0.001 |
| Relative difference pre-colonoscopy: Heart rate (bpm), median (IQR) | 7.0 (-2.0 - 18.0) | 6.0 (-2.0 - 18.0) | 0.42 | <0.001 |
| Relative difference post-colonoscopy: Hemoglobin (g/dL), median (IQR) | 0.3 (-0.7 - 1.2) | 0.3 (-1.0 - 1.6) | 0.39 | <0.001 |
| Relative difference post-colonoscopy: Mean corpuscular volume (fL), median (IQR) | -0.9 (-2.7 - 0.5) | -0.8 (-2.4 - 0.8) | 0.26 | <0.001 |
| Relative difference post-colonoscopy: Platelets (10^9/L), median (IQR) | 21.0 (-5.0 - 51.0) | 23.5 (-4.0 - 57.2) | 0.20 | <0.001 |
| Relative difference post-colonoscopy: Red cell distribution width (%), median (IQR) | -0.2 (-0.7 - 0.2) | -0.1 (-0.7 - 0.2) | 0.30 | <0.001 |
| Relative difference post-colonoscopy: Creatinine (mg/dL), median (IQR) | 0.1 (-0.0 - 0.3) | 0.1 (-0.0 - 0.3) | 0.43 | <0.1 |
| Relative difference post-colonoscopy: Glucose (mg/dL), median (IQR) | 6.5 (-11.5 - 32.2) | 9.0 (-13.0 - 37.0) | 0.26 | <0.001 |
| Relative difference post-colonoscopy: White blood cell count (10^9/L), median (IQR) | 0.9 (-0.6 - 2.4) | 1.2 (-0.4 - 3.0) | 0.06 | <0.001 |
| Relative difference post-colonoscopy: Blood urea nitrogen (mg/dL), median (IQR) | 6.0 (1.0 - 13.5) | 5.0 (1.0 - 12.0) | 0.45 | <0.1 |
| Relative difference post-colonoscopy: Potassium (mmol/L), median (IQR) | 0.1 (-0.2 - 0.5) | 0.2 (-0.2 - 0.5) | 0.18 | <0.001 |
| Relative difference post-colonoscopy: Sodium (mmol/L), median (IQR) | -2.0 (-4.0 - 1.0) | -2.0 (-4.0 - 1.0) | 0.31 | <0.1 |
| Relative difference post-colonoscopy: Chloride (mmol/L), median (IQR) | -3.0 (-6.2 - 0.0) | -3.0 (-6.0 - 0.0) | 0.39 | <0.1 |
| Relative difference post-colonoscopy: Diastolic blood pressure (mmHg), median (IQR) | 5.0 (-4.0 - 12.0) | 4.0 (-5.0 - 13.0) | 0.49 | <0.01 |
| Relative difference post-colonoscopy: Systolic blood pressure (mmHg), median (IQR) | 7.0 (-7.0 - 21.0) | 7.0 (-8.0 - 23.0) | 0.35 | <0.001 |
| Relative difference post-colonoscopy: Heart rate (bpm), median (IQR) | 7.0 (-2.0 - 18.0) | 6.0 (-2.0 - 18.0) | 0.42 | <0.001 |
| Hospitalization Characteristics | | | | |
| Admit Source: Self Referral, % (N) | 55.8% (139) | 58.4% (268) | 0.56 | <0.001 |
| Arrived in ED, % (N) | 83.9% (209) | 80.8% (371) | 0.36 | <0.1 |
| Was in an ICU at any point during hospitalization, % (N) | 6.0% (15) | 6.3% (29) | 0.99 | <0.001 |
| Hospitalization occurred during COVID-19 public health advisories, % (N) | 9.2% (23) | 8.7% (40) | 0.92 | <0.1 |
| Discharge Disposition: Home or Self-care, % (N) | 63.1% (157) | 62.7% (288) | 1.00 | <0.01 |
| Number of ICD10s associated with stay, mean (95% CI) | 21.6 (20.9 - 22.2) | 20.8 (20.4 - 21.2) | <0.05 | <0.1 |
| No complications occurred during hospitalization (based on MS-DRG), % (N) | 16.5% (41) | 17.2% (79) | 0.88 | <0.001 |
| Hospitalization related to: Dementia, % (N) | 1.6% (4) | 2.6% (12) | 0.55 | <0.001 |
| Hospitalization related to: GI Bleed, % (N) | 48.2% (120) | 45.1% (207) | 0.48 | <0.1 |
| Hospitalization related to: Cirrhosis, % (N) | 9.2% (23) | 9.2% (42) | 0.92 | <0.01 |
| Hospitalization related to: Diabetes, % (N) | 27.7% (69) | 28.3% (130) | 0.93 | <0.001 |
| Hospitalization related to: Kidney Disease, % (N) | 24.1% (60) | 23.7% (109) | 0.99 | <0.01 |
| Medication ordered before colonoscopy: Antihypertensive, % (N) | 4.8% (12) | 4.1% (19) | 0.82 | <0.1 |
| Medication ordered before colonoscopy: Heparin, % (N) | 4.8% (12) | 2.4% (11) | 0.13 | 0.13 |
| Medication ordered before colonoscopy: Opioid, % (N) | 11.6% (29) | 12.4% (57) | 0.86 | <0.001 |
| Medication ordered before colonoscopy: Tricyclic antidepressant, % (N) | 0.4% (1) | 0.7% (3) | 0.92 | <0.001 |
| Medication ordered before colonoscopy: Warfarin, % (N) | 1.2% (3) | 1.3% (6) | 0.81 | <0.001 |
| Medication ordered after colonoscopy: Antihypertensive, % (N) | 9.2% (23) | 8.5% (39) | 0.85 | <0.1 |
| Medication ordered after colonoscopy: Heparin, % (N) | 6.4% (16) | 6.3% (29) | 0.92 | <0.01 |
| Medication ordered after colonoscopy: Opioid, % (N) | 28.9% (72) | 29.2% (134) | 0.99 | <0.001 |
| Medication ordered after colonoscopy: Tricyclic antidepressant, % (N) | 1.6% (4) | 1.1% (5) | 0.81 | <0.1 |
| Medication ordered after colonoscopy: Warfarin, % (N) | 8.0% (20) | 6.5% (30) | 0.56 | <0.1 |
| Nothing by mouth or liquid diet before colonoscopy, % (N) | 100.0% (249) | 100.0% (459) | 1.00 | <0.001 |
| Number of all blood transfusions given during hospitalization, mean (95% CI) | 4.2 (3.9 - 4.4) | 3.8 (3.7 - 4.0) | <0.05 | <0.1 |
| Number of blood transfusions given before procedure, mean (95% CI) | 0.5 (0.4 - 0.6) | 0.4 (0.3 - 0.4) | <0.05 | <0.1 |
| Number of blood transfusions given after procedure, mean (95% CI) | 1.7 (1.5 - 1.9) | 1.7 (1.5 - 1.8) | 0.61 | <0.01 |
| Number of surgical procedures during hospitalization, mean (95% CI) | 2.5 (2.3 - 2.7) | 2.3 (2.2 - 2.5) | 0.11 | 0.11 |
| Number of surgical procedures between bowel preparation order and colonoscopy, mean (95% CI) | 0.0 (-0.003 - 0.01) | 0.0 (-0.001 - 0.01) | 0.95 | <0.001 |
| Number of surgical procedures after colonoscopy, mean (95% CI) | 0.3 (0.2 - 0.3) | 0.3 (0.2 - 0.3) | 0.53 | <0.1 |
| Colonoscopy Characteristics | | | | |
| Colonoscopy performed before noon (12 PM), % (N) | 33.7% (84) | 34.2% (157) | 0.97 | <0.001 |
| Same-day double exam: Colonoscopy and esophagogastroduodenoscopy, % (N) | 51.8% (129) | 47.1% (216) | 0.83 | <0.1 |
| Same-day double exam: Colonoscopy performed with any another endoscopy procedure, % (N) | 4.0% (10) | 3.7% (17) | 1.00 | <0.1 |
| American Society Of Anesthesiologists Rating, mean (95% CI) | 2.3 (2.1 - 2.5) | 2.3 (2.1 - 2.4) | 0.96 | <0.001 |
| Indication: Diarrhea, % (N) | 12.4% (28) | 12.7% (53) | 0.98 | <0.001 |
| Indication: Hematochezia, % (N) | 27.6% (62) | 24.0% (100) | 0.37 | <0.1 |
| Indication: Anemia, % (N) | 20.4% (46) | 18.0% (75) | 0.51 | <0.1 |
| Indication: Abnormal imaging, % (N) | 5.8% (13) | 8.4% (35) | 0.30 | <0.001 |
| Indication: Melena, % (N) | 8.4% (19) | 8.4% (35) | 0.90 | <0.01 |
| Indication: Inflammatory bowel disease, % (N) | 0.4% (1) | 0.2% (1) | 0.77 | <0.1 |
| Indication: Rectal bleeding, % (N) | 4.4% (10) | 4.3% (18) | 0.90 | <0.01 |
| Indication: Abdominal pain, % (N) | 2.2% (5) | 2.9% (12) | 0.81 | <0.001 |
| Indication: Other, % (N) | 18.7% (42) | 21.1% (88) | 0.53 | <0.001 |

**Supplemental Table 3. Predictors of hospital length of stay.** The adjusted association of covariates from a multivariate generalized linear model (N=704)**.**

| Variable | β (95% CI) | P Value |
| --- | --- | --- |
| Received low volume bowel preparation | -0.09 (-0.21 - 0.02) | 0.12 |
| 75 and above | -0.17 (-0.30 - -0.05) | <0.01 |
| Sex (Male) | 0.16 (0.04 - 0.27) | <0.01 |
| Arrived in ED | -0.23 (-0.37 - -0.08) | <0.01 |
| Admit Source: Self Referral | -0.01 (-0.16 - 0.13) | 0.85 |
| Discharge Disposition: Home or Self-care | -0.81 (-0.96 - -0.67) | <0.001 |
| Was in an ICU | 0.45 (0.32 - 0.58) | <0.001 |
| Hospitalization occurred during COVID-19 public health advisories | 0.00 (-0.19 - 0.20) | 0.98 |
| Number of ICD10s associated with stay | 0.48 (0.19 - 0.78) | <0.01 |
| No complications occurred during hospitalization (based on MS-DRG) | 0.28 (0.11 - 0.44) | <0.01 |
| Hospitalization related to: GI Bleed | -0.12 (-0.25 - 0.00) | <0.05 |
| Hospitalization related to: Kidney Disease | -0.05 (-0.17 - 0.08) | 0.49 |
| Medication ordered before colonoscopy: Opioid | 0.10 (-0.04 - 0.24) | 0.17 |
| Medication ordered after colonoscopy: Heparin | 0.08 (-0.07 - 0.23) | 0.31 |
| Medication ordered after colonoscopy: Opioid | 0.28 (0.16 - 0.39) | <0.001 |
| Cancer score | 0.20 (0.08 - 0.33) | <0.01 |
| Indication: Other | 0.34 (0.22 - 0.45) | <0.001 |

**Supplemental Table 4. Predictors of adequate bowel preparation (Aronchick scale 1 or 2).** The adjusted association of covariates from a multivariate generalized linear model (N=542)**.**

| Variable | Odds Ratio (95% CI) | P Value |
| --- | --- | --- |
| Received low volume bowel preparation | 1.02 (0.71 - 1.46) | 0.92 |
| 75 and above | 1.18 (0.78 - 1.78) | 0.42 |
| Discharge Disposition: Home or Self-care | 1.25 (0.84 - 1.85) | 0.27 |
| Was in an ICU at any point during hospitalization | 1.67 (0.74 - 3.77) | 0.22 |
| Hospitalization occurred during COVID-19 public health advisories | 0.73 (0.39 - 1.36) | 0.32 |
| No complications occurred during hospitalization (based on MS-DRG) | 1.18 (0.73 - 1.93) | 0.50 |
| Hospitalization related to: GI Bleed | 0.79 (0.55 - 1.13) | 0.20 |
| Hospitalization related to: Kidney Disease | 0.82 (0.55 - 1.24) | 0.35 |
| Medication ordered before colonoscopy: Opioid | 0.5 (0.3 - 0.85) | <0.01 |

**Supplementary Table 5. Baseline characteristics of the 1:2 propensity score matched** **GI bleed subpopulation**

^*^The number of missing variable values among patients receiving high volume preparation: Baseline lab measurements (N=1), Post procedure lab measurements (N=31); Colonoscopy Indication (N=9); Adequate bowel preparation score (N=35); Cecal intubation completed (N=27)

The number of missing variable values among patients receiving low volume preparation: Post procedure lab measurements (N=9); Colonoscopy Indication (N=9); Adequate bowel preparation score (N=22); Cecal intubation completed (N=21)

| Variable | Low volume, n = 102 | High volume, n = 177 | P-value | Standardized mean differences |
| --- | --- | --- | --- | --- |
| Patient Characteristics | | | | |
| 44 and below, % (N) | 7.8% (8) | 6.8% (12) | 0.93 | <0.1 |
| 45-59, % (N) | 15.7% (16) | 16.9% (30) | 0.92 | <0.001 |
| 60-74, % (N) | 38.2% (39) | 37.9% (67) | 0.95 | <0.1 |
| 75 and above, % (N) | 38.2% (39) | 38.4% (68) | 0.92 | <0.001 |
| Sex (Male), % (N) | 56.9% (58) | 62.1% (110) | 0.46 | <0.001 |
| BMI above 30, % (N) | 25.5% (26) | 26.6% (47) | 0.96 | <0.001 |
| Charlson Comorbidity Index, mean (95% CI) | 3.5 (3.2 - 3.9) | 3.5 (3.2 - 3.7) | 0.75 | <0.1 |
| AIDS/HIV score, % (N) | 1.0% (1) | 1.1% (2) | 0.91 | <0.001 |
| Cancer score, % (N) | 16.7% (17) | 14.7% (26) | 0.69 | <0.1 |
| Cerebrovascular disease score, % (N) | 8.8% (9) | 8.5% (15) | 0.92 | <0.1 |
| Chronic pulmonary disease score, % (N) | 25.5% (26) | 24.3% (43) | 0.85 | <0.1 |
| Congestive heart failure score, % (N) | 32.4% (33) | 32.8% (58) | 0.95 | <0.001 |
| Dementia score, % (N) | 3.9% (4) | 4.0% (7) | 0.99 | <0.001 |
| Diabetes score, % (N) | 36.3% (37) | 31.6% (56) | 0.52 | <0.1 |
| Hemiplegia/paraplegia score, % (N) | 0.0% (0) | 0.0% (0) | 1.00 | <0.001 |
| Liver disease score, % (N) | 15.7% (16) | 16.4% (29) | 0.89 | <0.001 |
| Myocardial infarction score, % (N) | 14.7% (15) | 14.1% (25) | 0.90 | <0.1 |
| Peptic ulcer disease score, % (N) | 18.6% (19) | 19.8% (35) | 0.83 | <0.001 |
| Peripheral vascular disease score, % (N) | 19.6% (20) | 20.9% (37) | 0.82 | <0.001 |
| Renal disease score, % (N) | 26.5% (27) | 29.4% (52) | 0.66 | <0.001 |
| Rheumatic disease score, % (N) | 6.9% (7) | 6.2% (11) | 0.84 | <0.1 |
| Baseline: Hemoglobin (g/dL), median (IQR) | 9.1 (7.4 - 10.7) | 9.3 (7.3 - 11.4) | 0.31 | <0.001 |
| Baseline: Mean corpuscular volume (fL), median (IQR) | 90.1 (85.4 - 94.9) | 91.7 (86.2 - 96.2) | 0.06 | <0.001 |
| Baseline: Platelets (10^9/L), median (IQR) | 217.5 (180.2 - 286.5) | 228.0 (164.0 - 283.0) | 0.44 | <0.001 |
| Baseline: Red cell distribution width (%), median (IQR) | 14.9 (13.6 - 17.3) | 15.2 (13.6 - 17.3) | 0.41 | <0.1 |
| Baseline: Creatinine (mg/dL), median (IQR) | 1.1 (0.8 - 1.5) | 1.1 (0.8 - 1.6) | 0.32 | <0.001 |
| Baseline: Glucose (mg/dL), median (IQR) | 120.0 (103.0 - 164.0) | 119.0 (104.0 - 149.2) | 0.34 | <0.1 |
| Baseline: White blood cell count (10^9/L), median (IQR) | 7.9 (5.9 - 11.0) | 8.3 (6.4 - 10.7) | 0.48 | <0.001 |
| Baseline: Blood urea nitrogen (mg/dL), median (IQR) | 20.0 (13.2 - 29.8) | 21.0 (14.0 - 34.0) | 0.21 | <0.001 |
| Baseline: Potassium (mmol/L), median (IQR) | 4.1 (3.8 - 4.5) | 4.2 (3.9 - 4.6) | 0.09 | <0.001 |
| Baseline: Sodium (mmol/L), median (IQR) | 139.0 (135.0 - 140.0) | 138.0 (136.0 - 140.0) | 0.46 | <0.001 |
| Baseline: Chloride (mmol/L), median (IQR) | 101.0 (97.0 - 104.8) | 101.0 (98.0 - 104.0) | 0.44 | <0.001 |
| Baseline: Diastolic blood pressure (mmHg), median (IQR) | 68.5 (63.0 - 76.0) | 68.0 (60.0 - 78.0) | 0.32 | <0.1 |
| Baseline: Systolic blood pressure (mmHg), median (IQR) | 136.0 (121.0 - 151.8) | 134.0 (120.0 - 149.0) | 0.31 | <0.1 |
| Baseline: Heart rate (bpm), median (IQR) | 82.0 (73.0 - 98.0) | 81.0 (70.0 - 94.0) | 0.14 | 0.11 |
| Relative difference pre-colonoscopy: Hemoglobin (g/dL), median (IQR) | 0.2 (-1.1 - 1.5) | 0.1 (-1.1 - 1.3) | 0.49 | <0.001 |
| Relative difference pre-colonoscopy: Mean corpuscular volume (fL), median (IQR) | -0.2 (-1.9 - 1.3) | 0.0 (-1.3 - 1.9) | 0.18 | <0.001 |
| Relative difference pre-colonoscopy: Platelets (10^9/L), median (IQR) | 19.0 (-1.8 - 47.0) | 24.0 (-3.0 - 50.0) | 0.30 | <0.001 |
| Relative difference pre-colonoscopy: Red cell distribution width (%), median (IQR) | -0.2 (-0.7 - 0.2) | -0.1 (-0.6 - 0.2) | 0.31 | <0.1 |
| Relative difference pre-colonoscopy: Creatinine (mg/dL), median (IQR) | 0.1 (0.0 - 0.3) | 0.1 (0.0 - 0.3) | 0.46 | <0.001 |
| Relative difference pre-colonoscopy: Glucose (mg/dL), median (IQR) | 18.0 (0.0 - 46.0) | 14.0 (0.0 - 44.2) | 0.28 | <0.001 |
| Relative difference pre-colonoscopy: White blood cell count (10^9/L), median (IQR) | 1.1 (-0.2 - 2.7) | 1.3 (-0.0 - 2.9) | 0.46 | <0.001 |
| Relative difference pre-colonoscopy: Blood urea nitrogen (mg/dL), median (IQR) | 6.0 (2.0 - 11.8) | 6.0 (1.0 - 14.0) | 0.41 | <0.1 |
| Relative difference pre-colonoscopy: Potassium (mmol/L), median (IQR) | 0.3 (-0.1 - 0.6) | 0.3 (-0.1 - 0.6) | 0.31 | <0.001 |
| Relative difference pre-colonoscopy: Sodium (mmol/L), median (IQR) | -2.0 (-4.0 - 0.0) | -2.0 (-5.0 - 0.0) | 0.31 | <0.1 |
| Relative difference pre-colonoscopy: Chloride (mmol/L), median (IQR) | -3.0 (-7.0 - 0.0) | -3.0 (-6.0 - 0.0) | 0.38 | <0.001 |
| Relative difference pre-colonoscopy: Diastolic blood pressure (mmHg), median (IQR) | 6.0 (-4.0 - 12.8) | 4.0 (-5.0 - 12.0) | 0.25 | <0.1 |
| Relative difference pre-colonoscopy: Systolic blood pressure (mmHg), median (IQR) | 6.0 (-5.8 - 17.8) | 5.0 (-10.0 - 23.0) | 0.37 | <0.1 |
| Relative difference pre-colonoscopy: Heart rate (bpm), median (IQR) | 8.0 (0.2 - 18.8) | 5.0 (-4.0 - 17.0) | 0.06 | 0.13 |
| Relative difference post-colonoscopy: Hemoglobin (g/dL), median (IQR) | 0.3 (-0.9 - 1.1) | 0.2 (-1.4 - 1.5) | 0.47 | <0.001 |
| Relative difference post-colonoscopy: Mean corpuscular volume (fL), median (IQR) | -0.9 (-2.7 - 1.4) | -0.3 (-2.3 - 1.7) | 0.16 | <0.001 |
| Relative difference post-colonoscopy: Platelets (10^9/L), median (IQR) | 17.0 (-3.0 - 42.0) | 28.0 (-2.2 - 57.0) | 0.08 | <0.001 |
| Relative difference post-colonoscopy: Red cell distribution width (%), median (IQR) | -0.3 (-0.8 - 0.1) | -0.2 (-1.0 - 0.2) | 0.33 | <0.1 |
| Relative difference post-colonoscopy: Creatinine (mg/dL), median (IQR) | 0.1 (-0.0 - 0.3) | 0.1 (-0.0 - 0.3) | 0.38 | <0.1 |
| Relative difference post-colonoscopy: Glucose (mg/dL), median (IQR) | 17.0 (-17.0 - 43.0) | 11.0 (-11.5 - 34.0) | 0.34 | <0.001 |
| Relative difference post-colonoscopy: White blood cell count (10^9/L), median (IQR) | 1.1 (-0.2 - 2.4) | 1.3 (-0.4 - 3.4) | 0.32 | <0.001 |
| Relative difference post-colonoscopy: Blood urea nitrogen (mg/dL), median (IQR) | 6.0 (1.0 - 15.0) | 6.0 (3.0 - 15.0) | 0.27 | <0.1 |
| Relative difference post-colonoscopy: Potassium (mmol/L), median (IQR) | 0.3 (-0.1 - 0.6) | 0.3 (-0.0 - 0.7) | 0.28 | <0.001 |
| Relative difference post-colonoscopy: Sodium (mmol/L), median (IQR) | -2.0 (-4.0 - 1.0) | -1.0 (-4.0 - 1.0) | 0.44 | <0.1 |
| Relative difference post-colonoscopy: Chloride (mmol/L), median (IQR) | -3.0 (-8.0 - 0.0) | -3.0 (-6.0 - 0.0) | 0.42 | <0.1 |
| Relative difference post-colonoscopy: Diastolic blood pressure (mmHg), median (IQR) | 6.0 (-4.0 - 12.8) | 4.0 (-5.0 - 12.0) | 0.25 | <0.1 |
| Relative difference post-colonoscopy: Systolic blood pressure (mmHg), median (IQR) | 6.0 (-5.8 - 17.8) | 5.0 (-10.0 - 23.0) | 0.37 | <0.1 |
| Relative difference post-colonoscopy: Heart rate (bpm), median (IQR) | 8.0 (0.2 - 18.8) | 5.0 (-4.0 - 17.0) | 0.06 | 0.13 |
| Hospitalization Characteristics | | | | |
| Admit Source: Self Referral, % (N) | 59.8% (61) | 59.9% (106) | 0.91 | <0.001 |
| Arrived in ED, % (N) | 87.3% (89) | 85.9% (152) | 0.89 | <0.1 |
| Was in an ICU at any point during hospitalization, % (N) | 5.9% (6) | 6.8% (12) | 0.97 | <0.001 |
| Hospitalization occurred during COVID-19 public health advisories, % (N) | 9.8% (10) | 7.3% (13) | 0.62 | <0.1 |
| Discharge Disposition: Home or Self-care, % (N) | 59.8% (61) | 57.6% (102) | 0.82 | <0.1 |
| Number of ICD10s associated with stay, mean (95% CI) | 22.5 (21.6 - 23.4) | 22.1 (21.4 - 22.8) | 0.54 | <0.1 |
| No complications occurred during hospitalization (based on MS-DRG), % (N) | 14.7% (15) | 15.8% (28) | 0.94 | <0.001 |
| Hospitalization related to: Dementia, % (N) | 3.9% (4) | 4.0% (7) | 0.76 | <0.001 |
| Hospitalization related to: GI Bleed, % (N) | 100.0% (102) | 100.0% (177) | 1.00 | <0.001 |
| Hospitalization related to: Cirrhosis, % (N) | 9.8% (10) | 10.2% (18) | 0.91 | <0.001 |
| Hospitalization related to: Diabetes, % (N) | 37.3% (38) | 32.2% (57) | 0.47 | 0.11 |
| Hospitalization related to: Kidney Disease, % (N) | 30.4% (31) | 31.1% (55) | 0.99 | <0.001 |
| Medication ordered before colonoscopy: Antihypertensive, % (N) | 2.9% (3) | 1.7% (3) | 0.79 | <0.1 |
| Medication ordered before colonoscopy: Heparin, % (N) | 4.9% (5) | 2.8% (5) | 0.57 | 0.11 |
| Medication ordered before colonoscopy: Opioid, % (N) | 8.8% (9) | 10.2% (18) | 0.88 | <0.001 |
| Medication ordered before colonoscopy: Tricyclic antidepressant, % (N) | 1.0% (1) | 0.0% (0) | 0.78 | 0.14 |
| Medication ordered before colonoscopy: Warfarin, % (N) | 2.0% (2) | 1.7% (3) | 0.76 | <0.1 |
| Medication ordered after colonoscopy: Antihypertensive, % (N) | 11.8% (12) | 15.8% (28) | 0.45 | <0.001 |
| Medication ordered after colonoscopy: Heparin, % (N) | 4.9% (5) | 6.8% (12) | 0.71 | <0.001 |
| Medication ordered after colonoscopy: Opioid, % (N) | 22.5% (23) | 19.2% (34) | 0.61 | <0.1 |
| Medication ordered after colonoscopy: Tricyclic antidepressant, % (N) | 0.0% (0) | 0.0% (0) | 1.00 | <0.001 |
| Medication ordered after colonoscopy: Warfarin, % (N) | 7.8% (8) | 7.3% (13) | 0.93 | <0.1 |
| Nothing by mouth or liquid diet before colonoscopy, % (N) | 100.0% (102) | 100.0% (177) | 1.00 | <0.001 |
| Number of all blood transfusions given during hospitalization, mean (95% CI) | 4.1 (3.7 - 4.5) | 5.6 (5.3 - 6.0) | <0.001 | <0.001 |
| Number of blood transfusions given before procedure, mean (95% CI) | 0.7 (0.6 - 0.9) | 1.1 (1.0 - 1.3) | <0.01 | <0.001 |
| Number of blood transfusions given after procedure, mean (95% CI) | 1.2 (1.0 - 1.4) | 1.4 (1.2 - 1.6) | 0.22 | <0.001 |
| Number of surgical procedures during hospitalization, mean (95% CI) | 2.6 (2.3 - 2.9) | 2.6 (2.4 - 2.8) | 0.87 | <0.1 |
| Number of surgical procedures between bowel preparation order and colonoscopy, mean (95% CI) | 0.0 (-0.009 - 0.02) | 0.0 (-0.005 - 0.01) | 0.70 | <0.1 |
| Number of surgical procedures after colonoscopy, mean (95% CI) | 0.3 (0.1 - 0.4) | 0.3 (0.2 - 0.4) | 0.74 | <0.001 |
| Colonoscopy Characteristics | | | | |
| Colonoscopy performed before noon (12 PM), % (N) | 32.4% (33) | 39.0% (69) | 0.33 | <0.001 |
| Same-day double exam: Colonoscopy and esophagogastroduodenoscopy, % (N) | 51.0% (52) | 46.3% (82) | 0.76 | <0.1 |
| Same-day double exam: Colonoscopy performed with any another endoscopy procedure, % (N) | 4.9% (5) | 2.3% (4) | 0.40 | 0.14 |
| American Society Of Anesthesiologists Rating, mean (95% CI) | 2.3 (2.0 - 2.6) | 2.4 (2.2 - 2.7) | 0.62 | <0.001 |
| Indication: Diarrhea, % (N) | 3.2% (3) | 3.6% (6) | 0.84 | <0.001 |
| Indication: Hematochezia, % (N) | 44.1% (41) | 44.6% (75) | 0.97 | <0.001 |
| Indication: Anemia, % (N) | 16.1% (15) | 16.1% (27) | 0.87 | <0.1 |
| Indication: Abnormal imaging, % (N) | 1.1% (1) | 0.6% (1) | 0.75 | <0.1 |
| Indication: Melena, % (N) | 15.1% (14) | 13.7% (23) | 0.91 | <0.1 |
| Indication: Inflammatory bowel disease, % (N) | 0.0% (0) | 0.0% (0) | 1.00 | <0.001 |
| Indication: Rectal bleeding, % (N) | 7.5% (7) | 8.9% (15) | 0.88 | <0.001 |
| Indication: Abdominal pain, % (N) | 1.1% (1) | 1.2% (2) | 0.60 | <0.001 |
| Indication: Other, % (N) | 11.8% (11) | 11.3% (19) | 0.94 | <0.1 |

**Supplementary Table 6. Baseline characteristics of the 1:2 propensity score matched kidney disease subpopulation**

^*^The number of missing variable values among patients receiving high volume preparation: Post procedure lab measurements (N=2); Colonoscopy Indication (N=2); Adequate bowel preparation score (N=12); Cecal intubation completed (N=4). The number of missing variable values among patients receiving low volume preparation: Colonoscopy Indication (N=2); Adequate bowel preparation score (N=5); Cecal intubation completed (N=9)

| Variable | Low volume, n = 34 | High volume, n = 55 | P-value | Standardized mean differences |
| --- | --- | --- | --- | --- |
| Patient Characteristics | | | | |
| 44 and below, % (N) | 2.9% (1) | 1.8% (1) | 0.70 | <0.1 |
| 45-59, % (N) | 14.7% (5) | 14.5% (8) | 0.77 | <0.1 |
| 60-74, % (N) | 44.1% (15) | 38.2% (21) | 0.74 | 0.12 |
| 75 and above, % (N) | 38.2% (13) | 45.5% (25) | 0.65 | <0.001 |
| Sex (Male), % (N) | 61.8% (21) | 50.9% (28) | 0.44 | 0.22 |
| BMI above 30, % (N) | 29.4% (10) | 30.9% (17) | 0.93 | <0.001 |
| Charlson Comorbidity Index, mean (95% CI) | 6.2 (5.4 - 7.0) | 5.5 (4.9 - 6.1) | 0.17 | 0.29 |
| AIDS/HIV score, % (N) | 0.0% (0) | 0.0% (0) | 1.00 | <0.001 |
| Cancer score, % (N) | 23.5% (8) | 14.5% (8) | 0.34 | 0.23 |
| Cerebrovascular disease score, % (N) | 5.9% (2) | 7.3% (4) | 0.81 | <0.001 |
| Chronic pulmonary disease score, % (N) | 35.3% (12) | 21.8% (12) | 0.24 | 0.30 |
| Congestive heart failure score, % (N) | 50.0% (17) | 54.5% (30) | 0.77 | <0.001 |
| Dementia score, % (N) | 5.9% (2) | 3.6% (2) | 0.63 | 0.10 |
| Diabetes score, % (N) | 64.7% (22) | 49.1% (27) | 0.34 | 0.32 |
| Hemiplegia/paraplegia score, % (N) | 0.0% (0) | 0.0% (0) | 1.00 | <0.001 |
| Liver disease score, % (N) | 11.8% (4) | 27.3% (15) | 0.14 | <0.001 |
| Myocardial infarction score, % (N) | 26.5% (9) | 25.5% (14) | 0.93 | <0.1 |
| Peptic ulcer disease score, % (N) | 23.5% (8) | 12.7% (7) | 0.24 | 0.28 |
| Peripheral vascular disease score, % (N) | 26.5% (9) | 21.8% (12) | 0.66 | 0.11 |
| Renal disease score, % (N) | 97.1% (33) | 94.5% (52) | 0.91 | 0.12 |
| Rheumatic disease score, % (N) | 5.9% (2) | 5.5% (3) | 0.93 | <0.1 |
| Baseline: Hemoglobin (g/dL), median (IQR) | 8.4 (7.3 - 9.5) | 8.9 (7.2 - 11.1) | 0.18 | <0.001 |
| Baseline: Mean corpuscular volume (fL), median (IQR) | 89.9 (84.3 - 93.0) | 91.2 (85.2 - 96.8) | 0.24 | <0.001 |
| Baseline: Platelets (10^9/L), median (IQR) | 214.0 (181.0 - 267.2) | 202.0 (161.5 - 272.0) | 0.28 | 0.22 |
| Baseline: Red cell distribution width (%), median (IQR) | 15.9 (14.4 - 17.3) | 16.5 (14.4 - 17.8) | 0.39 | <0.001 |
| Baseline: Creatinine (mg/dL), median (IQR) | 1.8 (1.5 - 2.7) | 1.7 (1.3 - 2.9) | 0.23 | <0.001 |
| Baseline: Glucose (mg/dL), median (IQR) | 135.5 (111.5 - 194.2) | 123.0 (105.0 - 147.0) | <0.05 | 0.45 |
| Baseline: White blood cell count (10^9/L), median (IQR) | 8.7 (6.7 - 11.1) | 8.9 (6.0 - 11.2) | 0.39 | <0.1 |
| Baseline: Blood urea nitrogen (mg/dL), median (IQR) | 33.0 (22.5 - 51.0) | 37.0 (23.5 - 52.0) | 0.36 | <0.001 |
| Baseline: Potassium (mmol/L), median (IQR) | 4.3 (3.9 - 4.8) | 4.2 (3.8 - 4.7) | 0.14 | 0.36 |
| Baseline: Sodium (mmol/L), median (IQR) | 136.5 (134.0 - 139.0) | 138.0 (134.5 - 140.0) | 0.31 | <0.001 |
| Baseline: Chloride (mmol/L), median (IQR) | 99.0 (96.0 - 102.0) | 100.0 (95.0 - 103.5) | 0.42 | <0.1 |
| Baseline: Diastolic blood pressure (mmHg), median (IQR) | 65.5 (58.5 - 71.5) | 66.0 (60.0 - 73.5) | 0.38 | <0.001 |
| Baseline: Systolic blood pressure (mmHg), median (IQR) | 140.0 (118.5 - 149.8) | 137.0 (114.0 - 153.5) | 0.48 | <0.1 |
| Baseline: Heart rate (bpm), median (IQR) | 82.0 (74.0 - 95.2) | 78.0 (69.0 - 91.0) | 0.20 | <0.1 |
| Relative difference pre-colonoscopy: Hemoglobin (g/dL), median (IQR) | 0.1 (-0.7 - 0.8) | -0.4 (-1.6 - 1.0) | 0.29 | <0.001 |
| Relative difference pre-colonoscopy: Mean corpuscular volume (fL), median (IQR) | -0.1 (-1.8 - 1.3) | -0.1 (-2.5 - 1.2) | 0.34 | <0.1 |
| Relative difference pre-colonoscopy: Platelets (10^9/L), median (IQR) | 23.5 (-4.8 - 42.0) | 4.0 (-17.0 - 38.0) | 0.10 | 0.22 |
| Relative difference pre-colonoscopy: Red cell distribution width (%), median (IQR) | 0.0 (-0.3 - 0.3) | -0.3 (-1.2 - 0.2) | <0.05 | <0.1 |
| Relative difference pre-colonoscopy: Creatinine (mg/dL), median (IQR) | 0.3 (0.1 - 0.5) | 0.2 (-0.0 - 0.4) | 0.13 | <0.1 |
| Relative difference pre-colonoscopy: Glucose (mg/dL), median (IQR) | 35.0 (3.2 - 63.8) | 17.0 (2.0 - 42.5) | 0.06 | 0.34 |
| Relative difference pre-colonoscopy: White blood cell count (10^9/L), median (IQR) | 1.1 (0.3 - 1.7) | 1.3 (-0.1 - 3.6) | 0.29 | <0.1 |
| Relative difference pre-colonoscopy: Blood urea nitrogen (mg/dL), median (IQR) | 9.0 (4.5 - 25.0) | 9.0 (2.0 - 18.0) | 0.30 | 0.12 |
| Relative difference pre-colonoscopy: Potassium (mmol/L), median (IQR) | 0.3 (0.1 - 0.6) | 0.1 (-0.2 - 0.7) | 0.06 | 0.47 |
| Relative difference pre-colonoscopy: Sodium (mmol/L), median (IQR) | -3.5 (-6.0 - 0.0) | -1.0 (-5.0 - 1.5) | 0.06 | <0.001 |
| Relative difference pre-colonoscopy: Chloride (mmol/L), median (IQR) | -4.0 (-6.0 - 0.0) | -2.0 (-5.0 - 1.0) | 0.14 | <0.001 |
| Relative difference pre-colonoscopy: Diastolic blood pressure (mmHg), median (IQR) | 6.0 (-3.0 - 10.5) | 3.0 (-4.0 - 8.5) | 0.21 | 0.16 |
| Relative difference pre-colonoscopy: Systolic blood pressure (mmHg), median (IQR) | 5.0 (-9.2 - 16.8) | 3.0 (-12.0 - 20.0) | 0.34 | 0.10 |
| Relative difference pre-colonoscopy: Heart rate (bpm), median (IQR) | 6.5 (-1.5 - 15.8) | 4.0 (-3.0 - 13.0) | 0.30 | <0.001 |
| Relative difference post-colonoscopy: Hemoglobin (g/dL), median (IQR) | 0.2 (-1.2 - 1.0) | -0.2 (-1.3 - 1.7) | 0.25 | <0.001 |
| Relative difference post-colonoscopy: Mean corpuscular volume (fL), median (IQR) | -0.4 (-2.4 - 2.3) | -0.7 (-2.5 - 1.4) | 0.37 | <0.1 |
| Relative difference post-colonoscopy: Platelets (10^9/L), median (IQR) | 22.0 (0.8 - 42.5) | 13.0 (-11.0 - 48.0) | 0.28 | <0.1 |
| Relative difference post-colonoscopy: Red cell distribution width (%), median (IQR) | 0.0 (-0.6 - 0.2) | -0.4 (-1.4 - 0.1) | 0.08 | <0.1 |
| Relative difference post-colonoscopy: Creatinine (mg/dL), median (IQR) | 0.3 (0.0 - 0.4) | 0.1 (-0.1 - 0.4) | 0.16 | <0.001 |
| Relative difference post-colonoscopy: Glucose (mg/dL), median (IQR) | 15.5 (-9.0 - 39.8) | 7.0 (-23.0 - 27.0) | 0.09 | 0.34 |
| Relative difference post-colonoscopy: White blood cell count (10^9/L), median (IQR) | 1.1 (0.1 - 2.1) | 1.2 (0.0 - 3.7) | 0.24 | <0.001 |
| Relative difference post-colonoscopy: Blood urea nitrogen (mg/dL), median (IQR) | 12.5 (2.2 - 23.0) | 10.0 (3.0 - 16.0) | 0.26 | 0.13 |
| Relative difference post-colonoscopy: Potassium (mmol/L), median (IQR) | 0.3 (-0.0 - 0.6) | 0.2 (-0.2 - 0.5) | 0.06 | 0.49 |
| Relative difference post-colonoscopy: Sodium (mmol/L), median (IQR) | -2.0 (-4.0 - 0.8) | -1.0 (-4.0 - 1.0) | 0.16 | <0.001 |
| Relative difference post-colonoscopy: Chloride (mmol/L), median (IQR) | -3.0 (-7.0 - 0.8) | -3.0 (-6.0 - 0.0) | 0.49 | <0.1 |
| Relative difference post-colonoscopy: Diastolic blood pressure (mmHg), median (IQR) | 6.0 (-3.0 - 10.5) | 3.0 (-4.0 - 8.5) | 0.21 | 0.16 |
| Relative difference post-colonoscopy: Systolic blood pressure (mmHg), median (IQR) | 5.0 (-9.2 - 16.8) | 3.0 (-12.0 - 20.0) | 0.34 | 0.10 |
| Relative difference post-colonoscopy: Heart rate (bpm), median (IQR) | 6.5 (-1.5 - 15.8) | 4.0 (-3.0 - 13.0) | 0.30 | <0.001 |
| Hospitalization Characteristics | | | | |
| Admit Source: Self Referral, % (N) | 44.1% (15) | 43.6% (24) | 0.86 | <0.1 |
| Arrived in ED, % (N) | 76.5% (26) | 74.5% (41) | 0.96 | <0.1 |
| Was in an ICU at any point during hospitalization, % (N) | 8.8% (3) | 5.5% (3) | 0.86 | 0.13 |
| Hospitalization occurred during COVID-19 public health advisories, % (N) | 2.9% (1) | 9.1% (5) | 0.49 | <0.001 |
| Discharge Disposition: Home or Self-care, % (N) | 38.2% (13) | 36.4% (20) | 0.96 | <0.1 |
| Number of ICD10s associated with stay, mean (95% CI) | 28.0 (26.2 - 29.8) | 26.8 (25.4 - 28.1) | 0.27 | 0.16 |
| No complications occurred during hospitalization (based on MS-DRG), % (N) | 8.8% (3) | 5.5% (3) | 0.86 | 0.13 |
| Hospitalization related to: Dementia, % (N) | 5.9% (2) | 3.6% (2) | 0.98 | 0.11 |
| Hospitalization related to: GI Bleed, % (N) | 55.9% (19) | 49.1% (27) | 0.81 | 0.14 |
| Hospitalization related to: Cirrhosis, % (N) | 8.8% (3) | 16.4% (9) | 0.49 | <0.001 |
| Hospitalization related to: Diabetes, % (N) | 67.6% (23) | 50.9% (28) | 0.18 | 0.35 |
| Hospitalization related to: Kidney Disease, % (N) | 100.0% (34) | 100.0% (55) | 1.00 | <0.001 |
| Medication ordered before colonoscopy: Antihypertensive, % (N) | 2.9% (1) | 0.0% (0) | 0.81 | 0.25 |
| Medication ordered before colonoscopy: Heparin, % (N) | 8.8% (3) | 3.6% (2) | 0.58 | 0.22 |
| Medication ordered before colonoscopy: Opioid, % (N) | 2.9% (1) | 5.5% (3) | 0.98 | <0.001 |
| Medication ordered before colonoscopy: Tricyclic antidepressant, % (N) | 0.0% (0) | 0.0% (0) | 1.00 | <0.001 |
| Medication ordered before colonoscopy: Warfarin, % (N) | 2.9% (1) | 5.5% (3) | 0.98 | <0.001 |
| Medication ordered after colonoscopy: Antihypertensive, % (N) | 17.6% (6) | 20.0% (11) | 1.00 | <0.001 |
| Medication ordered after colonoscopy: Heparin, % (N) | 8.8% (3) | 7.3% (4) | 0.89 | <0.1 |
| Medication ordered after colonoscopy: Opioid, % (N) | 26.5% (9) | 29.1% (16) | 0.98 | <0.001 |
| Medication ordered after colonoscopy: Tricyclic antidepressant, % (N) | 0.0% (0) | 0.0% (0) | 1.00 | <0.001 |
| Medication ordered after colonoscopy: Warfarin, % (N) | 11.8% (4) | 14.5% (8) | 0.96 | <0.001 |
| Nothing by mouth or liquid diet before colonoscopy, % (N) | 100.0% (34) | 100.0% (55) | 1.00 | <0.001 |
| Number of all blood transfusions given during hospitalization, mean (95% CI) | 4.8 (4.1 - 5.5) | 6.1 (5.5 - 6.8) | <0.01 | <0.001 |
| Number of blood transfusions given before procedure, mean (95% CI) | 0.6 (0.3 - 0.8) | 0.5 (0.4 - 0.7) | 0.79 | <0.1 |
| Number of blood transfusions given after procedure, mean (95% CI) | 1.6 (1.2 - 2.0) | 2.5 (2.1 - 2.9) | <0.01 | <0.001 |
| Number of surgical procedures during hospitalization, mean (95% CI) | 2.8 (2.2 - 3.3) | 2.7 (2.3 - 3.2) | 0.92 | <0.1 |
| Number of surgical procedures between bowel preparation order and colonoscopy, mean (95% CI) | 0.0 (0 - 0) | 0.0 (-0.01 - 0.05) | 1.00 | <0.001 |
| Number of surgical procedures after colonoscopy, mean (95% CI) | 0.3 (0.09 - 0.4) | 0.3 (0.1 - 0.4) | 0.82 | <0.001 |
| Colonoscopy Characteristics | | | | |
| Colonoscopy performed before noon (12 PM), % (N) | 29.4% (10) | 40.0% (22) | 0.43 | <0.001 |
| Same-day double exam: Colonoscopy and esophagogastroduodenoscopy, % (N) | 61.8% (21) | 60.0% (33) | 0.95 | <0.1 |
| Same-day double exam: Colonoscopy performed with any another endoscopy procedure, % (N) | 8.8% (3) | 3.6% (2) | 0.58 | 0.22 |
| American Society Of Anesthesiologists Rating, mean (95% CI) | 2.5 (2.0 - 3.1) | 2.6 (2.2 - 3.0) | 0.88 | <0.001 |
| Indication: Diarrhea, % (N) | 6.3% (2) | 17.0% (9) | 0.27 | <0.001 |
| Indication: Hematochezia, % (N) | 12.5% (4) | 15.1% (8) | 0.99 | <0.001 |
| Indication: Anemia, % (N) | 40.6% (13) | 30.2% (16) | 0.46 | 0.22 |
| Indication: Abnormal imaging, % (N) | 6.3% (2) | 0.0% (0) | 0.27 | 0.37 |
| Indication: Melena, % (N) | 15.6% (5) | 17.0% (9) | 0.89 | <0.001 |
| Indication: Inflammatory bowel disease, % (N) | 0.0% (0) | 0.0% (0) | 1.00 | <0.001 |
| Indication: Rectal bleeding, % (N) | 3.1% (1) | 1.9% (1) | 0.71 | <0.1 |
| Indication: Abdominal pain, % (N) | 3.1% (1) | 5.7% (3) | 1.00 | <0.001 |
| Indication: Other, % (N) | 12.5% (4) | 13.2% (7) | 0.81 | <0.001 |

**Supplementary Table 7. Baseline characteristics of the 1:2 propensity score matched subpopulation of patients** **that received opioids prior to the colonoscopy**

^*^The number of missing variable values among patients receiving high volume preparation: Post procedure lab measurements (N=3); Colonoscopy Indication (N=1); Adequate bowel preparation score (N=7); Cecal intubation completed (N=6). The number of missing variable values among patients receiving low volume preparation: Post procedure lab measurements (N=3); Colonoscopy Indication (N=1); Adequate bowel preparation score (N=4); Cecal intubation completed (N=2)

| Variable | Low volume, n = 20 | High volume, n = 33 | P-value | Standardized mean differences |
| --- | --- | --- | --- | --- |
| Patient Characteristics | | | | |
| 44 and below, % (N) | 35.0% (7) | 36.4% (12) | 0.85 | <0.001 |
| 45-59, % (N) | 30.0% (6) | 30.3% (10) | 0.78 | <0.001 |
| 60-74, % (N) | 25.0% (5) | 24.2% (8) | 0.79 | <0.1 |
| 75 and above, % (N) | 10.0% (2) | 9.1% (3) | 0.71 | <0.1 |
| Sex (Male), % (N) | 65.0% (13) | 63.6% (21) | 0.85 | <0.1 |
| BMI above 30, % (N) | 20.0% (4) | 18.2% (6) | 0.84 | <0.1 |
| Charlson Comorbidity Index, mean (95% CI) | 2.7 (1.9 - 3.4) | 3.2 (2.6 - 3.9) | 0.23 | <0.001 |
| AIDS/HIV score, % (N) | 0.0% (0) | 6.1% (2) | 1.00 | <0.001 |
| Cancer score, % (N) | 20.0% (4) | 18.2% (6) | 0.88 | <0.1 |
| Cerebrovascular disease score, % (N) | 0.0% (0) | 3.0% (1) | 1.00 | <0.001 |
| Chronic pulmonary disease score, % (N) | 30.0% (6) | 27.3% (9) | 0.86 | <0.1 |
| Congestive heart failure score, % (N) | 0.0% (0) | 24.2% (8) | 1.00 | <0.001 |
| Dementia score, % (N) | 0.0% (0) | 0.0% (0) | 1.00 | <0.001 |
| Diabetes score, % (N) | 10.0% (2) | 15.2% (5) | 0.62 | <0.001 |
| Hemiplegia/paraplegia score, % (N) | 0.0% (0) | 3.0% (1) | 1.00 | <0.001 |
| Liver disease score, % (N) | 30.0% (6) | 24.2% (8) | 0.69 | 0.13 |
| Myocardial infarction score, % (N) | 5.0% (1) | 15.2% (5) | 0.31 | <0.001 |
| Peptic ulcer disease score, % (N) | 5.0% (1) | 15.2% (5) | 0.31 | <0.001 |
| Peripheral vascular disease score, % (N) | 10.0% (2) | 9.1% (3) | 0.92 | <0.1 |
| Renal disease score, % (N) | 15.0% (3) | 12.1% (4) | 0.78 | <0.1 |
| Rheumatic disease score, % (N) | 15.0% (3) | 6.1% (2) | 0.32 | 0.29 |
| Baseline: Hemoglobin (g/dL), median (IQR) | 11.7 (9.0 - 13.2) | 10.2 (9.1 - 12.1) | 0.16 | 0.23 |
| Baseline: Mean corpuscular volume (fL), median (IQR) | 88.8 (86.4 - 94.8) | 86.7 (82.6 - 90.8) | 0.12 | 0.30 |
| Baseline: Platelets (10^9/L), median (IQR) | 268.0 (210.5 - 300.5) | 254.0 (221.0 - 324.0) | 0.32 | <0.001 |
| Baseline: Red cell distribution width (%), median (IQR) | 14.8 (13.6 - 15.9) | 15.4 (14.2 - 17.1) | 0.12 | <0.001 |
| Baseline: Creatinine (mg/dL), median (IQR) | 0.9 (0.6 - 1.1) | 0.9 (0.7 - 1.0) | 0.35 | <0.001 |
| Baseline: Glucose (mg/dL), median (IQR) | 108.0 (100.0 - 118.0) | 104.0 (92.0 - 133.0) | 0.44 | <0.001 |
| Baseline: White blood cell count (10^9/L), median (IQR) | 7.1 (4.6 - 10.4) | 8.7 (6.7 - 10.0) | 0.11 | <0.001 |
| Baseline: Blood urea nitrogen (mg/dL), median (IQR) | 11.5 (8.8 - 14.2) | 16.0 (9.0 - 20.0) | 0.05 | <0.001 |
| Baseline: Potassium (mmol/L), median (IQR) | 3.9 (3.6 - 4.1) | 4.1 (3.8 - 4.3) | <0.05 | <0.001 |
| Baseline: Sodium (mmol/L), median (IQR) | 138.5 (135.0 - 140.2) | 138.0 (135.0 - 141.0) | 0.50 | <0.001 |
| Baseline: Chloride (mmol/L), median (IQR) | 98.5 (97.0 - 103.5) | 101.0 (97.0 - 104.0) | 0.41 | <0.1 |
| Baseline: Diastolic blood pressure (mmHg), median (IQR) | 67.0 (63.8 - 72.2) | 76.0 (70.0 - 82.0) | <0.05 | <0.001 |
| Baseline: Systolic blood pressure (mmHg), median (IQR) | 127.5 (120.5 - 139.0) | 131.0 (117.0 - 147.0) | 0.31 | <0.001 |
| Baseline: Heart rate (bpm), median (IQR) | 89.0 (76.0 - 96.5) | 85.0 (75.0 - 95.0) | 0.36 | 0.19 |
| Relative difference pre-colonoscopy: Hemoglobin (g/dL), median (IQR) | 0.8 (0.1 - 1.6) | 0.4 (-0.9 - 2.1) | 0.39 | 0.12 |
| Relative difference pre-colonoscopy: Mean corpuscular volume (fL), median (IQR) | -1.4 (-2.1 - -0.0) | -1.2 (-4.9 - 0.6) | 0.32 | 0.33 |
| Relative difference pre-colonoscopy: Platelets (10^9/L), median (IQR) | 21.0 (-22.0 - 52.0) | 36.0 (1.0 - 52.0) | 0.30 | <0.001 |
| Relative difference pre-colonoscopy: Red cell distribution width (%), median (IQR) | -0.1 (-0.3 - 0.1) | -0.1 (-0.4 - 0.2) | 0.38 | 0.30 |
| Relative difference pre-colonoscopy: Creatinine (mg/dL), median (IQR) | 0.1 (0.0 - 0.3) | 0.1 (-0.0 - 0.2) | 0.12 | <0.001 |
| Relative difference pre-colonoscopy: Glucose (mg/dL), median (IQR) | 14.5 (1.2 - 33.2) | 13.0 (-8.0 - 24.0) | 0.40 | <0.001 |
| Relative difference pre-colonoscopy: White blood cell count (10^9/L), median (IQR) | 0.9 (0.5 - 1.6) | 0.7 (-1.0 - 2.6) | 0.31 | 0.17 |
| Relative difference pre-colonoscopy: Blood urea nitrogen (mg/dL), median (IQR) | 4.0 (0.8 - 6.2) | 3.0 (0.0 - 9.0) | 0.43 | <0.1 |
| Relative difference pre-colonoscopy: Potassium (mmol/L), median (IQR) | 0.0 (-0.4 - 0.5) | 0.3 (0.0 - 0.5) | 0.09 | <0.001 |
| Relative difference pre-colonoscopy: Sodium (mmol/L), median (IQR) | -2.0 (-4.0 - 1.2) | -1.0 (-3.0 - 1.0) | 0.22 | <0.001 |
| Relative difference pre-colonoscopy: Chloride (mmol/L), median (IQR) | -3.0 (-7.0 - -0.5) | -3.0 (-6.0 - 1.0) | 0.24 | <0.001 |
| Relative difference pre-colonoscopy: Diastolic blood pressure (mmHg), median (IQR) | 1.0 (-6.0 - 14.2) | 5.0 (-2.0 - 15.0) | 0.24 | <0.001 |
| Relative difference pre-colonoscopy: Systolic blood pressure (mmHg), median (IQR) | 3.5 (-7.2 - 15.8) | 6.0 (-10.0 - 17.0) | 0.50 | <0.001 |
| Relative difference pre-colonoscopy: Heart rate (bpm), median (IQR) | 9.5 (-0.8 - 26.2) | 5.0 (-1.0 - 14.0) | 0.16 | 0.25 |
| Relative difference post-colonoscopy: Hemoglobin (g/dL), median (IQR) | 0.6 (-0.2 - 1.1) | 0.5 (-0.7 - 1.9) | 0.50 | <0.1 |
| Relative difference post-colonoscopy: Mean corpuscular volume (fL), median (IQR) | -1.7 (-2.4 - -0.0) | -1.2 (-4.2 - -0.0) | 0.33 | 0.33 |
| Relative difference post-colonoscopy: Platelets (10^9/L), median (IQR) | 10.0 (-25.0 - 42.0) | 28.5 (-0.8 - 51.5) | 0.11 | <0.001 |
| Relative difference post-colonoscopy: Red cell distribution width (%), median (IQR) | -0.3 (-0.7 - 0.1) | 0.0 (-0.6 - 0.4) | 0.10 | <0.1 |
| Relative difference post-colonoscopy: Creatinine (mg/dL), median (IQR) | 0.0 (-0.0 - 0.2) | 0.0 (-0.0 - 0.1) | 0.24 | <0.001 |
| Relative difference post-colonoscopy: Glucose (mg/dL), median (IQR) | -1.0 (-19.0 - 11.2) | 2.0 (-13.0 - 18.5) | 0.28 | <0.001 |
| Relative difference post-colonoscopy: White blood cell count (10^9/L), median (IQR) | 1.1 (-0.3 - 2.7) | 1.3 (-0.5 - 2.9) | 0.50 | 0.15 |
| Relative difference post-colonoscopy: Blood urea nitrogen (mg/dL), median (IQR) | 2.0 (1.0 - 8.0) | 4.0 (-2.0 - 8.5) | 0.50 | <0.1 |
| Relative difference post-colonoscopy: Potassium (mmol/L), median (IQR) | 0.1 (-0.5 - 0.6) | 0.1 (-0.2 - 0.6) | 0.20 | <0.001 |
| Relative difference post-colonoscopy: Sodium (mmol/L), median (IQR) | -2.0 (-3.8 - 2.0) | -2.0 (-4.0 - 0.0) | 0.23 | 0.12 |
| Relative difference post-colonoscopy: Chloride (mmol/L), median (IQR) | -3.0 (-7.5 - 0.0) | -3.0 (-8.0 - -0.5) | 0.47 | <0.1 |
| Relative difference post-colonoscopy: Diastolic blood pressure (mmHg), median (IQR) | 1.0 (-6.0 - 14.2) | 5.0 (-2.0 - 15.0) | 0.24 | <0.001 |
| Relative difference post-colonoscopy: Systolic blood pressure (mmHg), median (IQR) | 3.5 (-7.2 - 15.8) | 6.0 (-10.0 - 17.0) | 0.50 | <0.001 |
| Relative difference post-colonoscopy: Heart rate (bpm), median (IQR) | 9.5 (-0.8 - 26.2) | 5.0 (-1.0 - 14.0) | 0.16 | 0.25 |
| Hospitalization Characteristics | | | | |
| Admit Source: Self Referral, % (N) | 75.0% (15) | 57.6% (19) | 0.32 | 0.38 |
| Arrived in ED, % (N) | 100.0% (20) | 93.9% (31) | <0.001 | 0.36 |
| Was in an ICU at any point during hospitalization, % (N) | 10.0% (2) | 6.1% (2) | 0.99 | 0.15 |
| Hospitalization occurred during COVID-19 public health advisories, % (N) | 5.0% (1) | 12.1% (4) | 0.71 | <0.001 |
| Discharge Disposition: Home or Self-care, % (N) | 70.0% (14) | 69.7% (23) | 0.78 | <0.1 |
| Number of ICD10s associated with stay, mean (95% CI) | 18.6 (16.6 - 20.5) | 21.7 (20.1 - 23.3) | <0.05 | <0.001 |
| No complications occurred during hospitalization (based on MS-DRG), % (N) | 10.0% (2) | 9.1% (3) | 0.71 | <0.1 |
| Hospitalization related to: Dementia, % (N) | 0.0% (0) | 0.0% (0) | 1.00 | <0.001 |
| Hospitalization related to: GI Bleed, % (N) | 45.0% (9) | 54.5% (18) | 0.80 | <0.001 |
| Hospitalization related to: Cirrhosis, % (N) | 10.0% (2) | 9.1% (3) | 0.71 | <0.1 |
| Hospitalization related to: Diabetes, % (N) | 10.0% (2) | 15.2% (5) | 0.91 | <0.001 |
| Hospitalization related to: Kidney Disease, % (N) | 5.0% (1) | 12.1% (4) | 0.71 | <0.001 |
| Medication ordered before colonoscopy: Antihypertensive, % (N) | 0.0% (0) | 6.1% (2) | 0.71 | <0.001 |
| Medication ordered before colonoscopy: Heparin, % (N) | 0.0% (0) | 6.1% (2) | 0.71 | <0.001 |
| Medication ordered before colonoscopy: Opioid, % (N) | 100.0% (20) | 100.0% (33) | 1.00 | <0.001 |
| Medication ordered before colonoscopy: Tricyclic antidepressant, % (N) | 0.0% (0) | 3.0% (1) | 0.80 | <0.001 |
| Medication ordered before colonoscopy: Warfarin, % (N) | 0.0% (0) | 0.0% (0) | 1.00 | <0.001 |
| Medication ordered after colonoscopy: Antihypertensive, % (N) | 5.0% (1) | 12.1% (4) | 0.71 | <0.001 |
| Medication ordered after colonoscopy: Heparin, % (N) | 0.0% (0) | 3.0% (1) | 0.80 | <0.001 |
| Medication ordered after colonoscopy: Opioid, % (N) | 50.0% (10) | 60.6% (20) | 0.64 | <0.001 |
| Medication ordered after colonoscopy: Tricyclic antidepressant, % (N) | 5.0% (1) | 9.1% (3) | 0.99 | <0.001 |
| Medication ordered after colonoscopy: Warfarin, % (N) | 0.0% (0) | 0.0% (0) | 1.00 | <0.001 |
| Nothing by mouth or liquid diet before colonoscopy, % (N) | 100.0% (20) | 100.0% (33) | 1.00 | <0.001 |
| Number of all blood transfusions given during hospitalization, mean (95% CI) | 3.6 (2.7 - 4.4) | 3.1 (2.5 - 3.7) | 0.34 | <0.1 |
| Number of blood transfusions given before procedure, mean (95% CI) | 0.7 (0.3 - 1.1) | 0.6 (0.3 - 0.9) | 0.68 | <0.1 |
| Number of blood transfusions given after procedure, mean (95% CI) | 0.4 (0.1 - 0.7) | 1.0 (0.6 - 1.3) | <0.05 | <0.001 |
| Number of surgical procedures during hospitalization, mean (95% CI) | 3.0 (2.2 - 3.7) | 2.9 (2.4 - 3.5) | 0.98 | <0.1 |
| Number of surgical procedures between bowel preparation order and colonoscopy, mean (95% CI) | 0.0 (-1) | 0.0 (-1) | 1.00 | <0.001 |
| Number of surgical procedures after colonoscopy, mean (95% CI) | 0.3 (0.03 - 0.4) | 0.4 (0.2 - 0.6) | 0.39 | <0.001 |
| Colonoscopy Characteristics | | | | |
| Colonoscopy performed before noon (12 PM), % (N) | 35.0% (7) | 54.5% (18) | 0.65 | <0.001 |
| Same-day double exam: Colonoscopy and esophagogastroduodenoscopy, % (N) | 35.0% (7) | 54.5% (18) | 0.65 | <0.001 |
| Same-day double exam: Colonoscopy performed with any another endoscopy procedure, % (N) | 5.0% (1) | 6.1% (2) | 0.65 | <0.001 |
| American Society Of Anesthesiologists Rating, mean (95% CI) | 2.3 (1.6 - 2.9) | 2.3 (1.8 - 2.8) | 0.90 | <0.001 |
| Indication: Diarrhea, % (N) | 15.8% (3) | 6.3% (2) | 0.54 | 0.31 |
| Indication: Hematochezia, % (N) | 15.8% (3) | 25.0% (8) | 0.67 | <0.001 |
| Indication: Anemia, % (N) | 15.8% (3) | 9.4% (3) | 0.81 | 0.19 |
| Indication: Abnormal imaging, % (N) | 5.3% (1) | 12.5% (4) | 0.72 | <0.001 |
| Indication: Melena, % (N) | 5.3% (1) | 6.3% (2) | 0.64 | <0.001 |
| Indication: Inflammatory bowel disease, % (N) | 0.0% (0) | 0.0% (0) | 1.00 | <0.001 |
| Indication: Rectal bleeding, % (N) | 0.0% (0) | 3.1% (1) | 0.79 | <0.001 |
| Indication: Abdominal pain, % (N) | 5.3% (1) | 12.5% (4) | 0.72 | <0.001 |
| Indication: Other, % (N) | 36.8% (7) | 25.0% (8) | 0.56 | 0.26 |

**Supplementary Table 8. Baseline characteristics of the 1:2 propensity score** **matched subpopulation of patients age 75 and above.**

^*^The number of missing variable values among patients receiving high volume preparation: Post procedure lab measurements (N=6); Colonoscopy Indication (N=6); Adequate bowel preparation score (N=23); Cecal intubation completed (N=11). The number of missing variable values among patients receiving low volume preparation: Post procedure lab measurements (N=4); Colonoscopy Indication (N=6); Adequate bowel preparation score (N=14); Cecal intubation completed (N=14)

| Variable | Low volume, n = 50 | High volume, n = 86 | P-value | Standardized mean differences |
| --- | --- | --- | --- | --- |
| Patient Characteristics | | | | |
| 44 and below, % (N) | 0.0% (0) | 0.0% (0) | 1.00 | <0.001 |
| 45-59, % (N) | 0.0% (0) | 0.0% (0) | 1.00 | <0.001 |
| 60-74, % (N) | 0.0% (0) | 0.0% (0) | 1.00 | <0.001 |
| 75 and above, % (N) | 100.0% (50) | 100.0% (86) | 1.00 | <0.001 |
| Sex (Male), % (N) | 54.0% (27) | 60.5% (52) | 0.58 | <0.001 |
| BMI above 30, % (N) | 20.0% (10) | 19.8% (17) | 0.85 | <0.1 |
| Charlson Comorbidity Index, mean (95% CI) | 3.5 (3.0 - 4.0) | 3.8 (3.4 - 4.2) | 0.41 | <0.001 |
| AIDS/HIV score, % (N) | 0.0% (0) | 0.0% (0) | 1.00 | <0.001 |
| Cancer score, % (N) | 18.0% (9) | 16.3% (14) | 0.81 | <0.1 |
| Cerebrovascular disease score, % (N) | 6.0% (3) | 7.0% (6) | 0.83 | <0.001 |
| Chronic pulmonary disease score, % (N) | 22.0% (11) | 27.9% (24) | 0.51 | <0.001 |
| Congestive heart failure score, % (N) | 44.0% (22) | 41.9% (36) | 0.85 | <0.1 |
| Dementia score, % (N) | 6.0% (3) | 2.3% (2) | 0.30 | 0.18 |
| Diabetes score, % (N) | 32.0% (16) | 36.0% (31) | 0.70 | <0.001 |
| Hemiplegia/paraplegia score, % (N) | 0.0% (0) | 0.0% (0) | 1.00 | <0.001 |
| Liver disease score, % (N) | 6.0% (3) | 9.3% (8) | 0.52 | <0.001 |
| Myocardial infarction score, % (N) | 16.0% (8) | 20.9% (18) | 0.53 | <0.001 |
| Peptic ulcer disease score, % (N) | 12.0% (6) | 16.3% (14) | 0.53 | <0.001 |
| Peripheral vascular disease score, % (N) | 22.0% (11) | 24.4% (21) | 0.78 | <0.001 |
| Renal disease score, % (N) | 38.0% (19) | 43.0% (37) | 0.66 | <0.001 |
| Rheumatic disease score, % (N) | 12.0% (6) | 11.6% (10) | 0.95 | <0.1 |
| Baseline: Hemoglobin (g/dL), median (IQR) | 9.1 (7.4 - 11.0) | 8.4 (6.9 - 10.6) | 0.15 | 0.17 |
| Baseline: Mean corpuscular volume (fL), median (IQR) | 92.2 (87.1 - 98.3) | 91.2 (85.5 - 97.0) | 0.24 | <0.1 |
| Baseline: Platelets (10^9/L), median (IQR) | 217.0 (176.5 - 253.2) | 243.0 (184.2 - 306.5) | 0.08 | <0.001 |
| Baseline: Red cell distribution width (%), median (IQR) | 15.0 (13.3 - 17.3) | 15.9 (14.4 - 17.9) | 0.12 | <0.001 |
| Baseline: Creatinine (mg/dL), median (IQR) | 1.1 (0.9 - 1.5) | 1.1 (0.9 - 1.5) | 0.33 | <0.001 |
| Baseline: Glucose (mg/dL), median (IQR) | 120.0 (104.0 - 163.0) | 121.0 (104.0 - 145.5) | 0.42 | <0.001 |
| Baseline: White blood cell count (10^9/L), median (IQR) | 8.3 (5.9 - 11.0) | 8.8 (6.2 - 11.0) | 0.23 | <0.001 |
| Baseline: Blood urea nitrogen (mg/dL), median (IQR) | 21.0 (16.0 - 34.8) | 25.0 (19.0 - 38.5) | 0.09 | <0.001 |
| Baseline: Potassium (mmol/L), median (IQR) | 4.1 (3.8 - 4.5) | 4.2 (4.0 - 4.6) | 0.12 | <0.001 |
| Baseline: Sodium (mmol/L), median (IQR) | 139.0 (135.2 - 141.0) | 138.0 (135.2 - 140.8) | 0.38 | <0.001 |
| Baseline: Chloride (mmol/L), median (IQR) | 102.0 (98.0 - 104.0) | 101.0 (97.2 - 104.0) | 0.37 | <0.001 |
| Baseline: Diastolic blood pressure (mmHg), median (IQR) | 67.0 (60.2 - 77.0) | 67.0 (62.2 - 75.0) | 0.48 | <0.001 |
| Baseline: Systolic blood pressure (mmHg), median (IQR) | 141.5 (122.5 - 155.0) | 142.0 (125.2 - 158.8) | 0.40 | <0.001 |
| Baseline: Heart rate (bpm), median (IQR) | 76.0 (68.5 - 87.2) | 74.0 (64.2 - 87.8) | 0.20 | 0.15 |
| Relative difference pre-colonoscopy: Hemoglobin (g/dL), median (IQR) | 0.6 (-1.4 - 1.6) | -0.3 (-1.7 - 0.9) | 0.09 | 0.15 |
| Relative difference pre-colonoscopy: Mean corpuscular volume (fL), median (IQR) | 0.4 (-1.4 - 2.0) | 0.3 (-2.0 - 1.8) | 0.31 | <0.001 |
| Relative difference pre-colonoscopy: Platelets (10^9/L), median (IQR) | 20.0 (0.8 - 43.0) | 21.5 (-3.8 - 58.0) | 0.30 | <0.001 |
| Relative difference pre-colonoscopy: Red cell distribution width (%), median (IQR) | -0.2 (-0.7 - 0.1) | -0.2 (-0.9 - 0.2) | 0.36 | 0.14 |
| Relative difference pre-colonoscopy: Creatinine (mg/dL), median (IQR) | 0.1 (0.1 - 0.3) | 0.2 (0.0 - 0.3) | 0.49 | 0.15 |
| Relative difference pre-colonoscopy: Glucose (mg/dL), median (IQR) | 18.0 (0.0 - 41.0) | 22.0 (7.0 - 39.8) | 0.24 | <0.001 |
| Relative difference pre-colonoscopy: White blood cell count (10^9/L), median (IQR) | 1.0 (-0.0 - 2.6) | 1.4 (0.1 - 3.1) | 0.23 | <0.001 |
| Relative difference pre-colonoscopy: Blood urea nitrogen (mg/dL), median (IQR) | 7.0 (3.2 - 12.0) | 8.0 (4.0 - 15.0) | 0.34 | <0.1 |
| Relative difference pre-colonoscopy: Potassium (mmol/L), median (IQR) | 0.3 (-0.1 - 0.5) | 0.2 (-0.1 - 0.7) | 0.38 | <0.001 |
| Relative difference pre-colonoscopy: Sodium (mmol/L), median (IQR) | -2.0 (-4.0 - 0.0) | -1.0 (-4.0 - 0.8) | 0.20 | <0.001 |
| Relative difference pre-colonoscopy: Chloride (mmol/L), median (IQR) | -4.0 (-7.0 - 0.0) | -3.0 (-5.0 - 0.0) | 0.14 | <0.001 |
| Relative difference pre-colonoscopy: Diastolic blood pressure (mmHg), median (IQR) | 4.0 (-3.0 - 13.8) | 5.0 (-3.0 - 13.0) | 0.44 | <0.1 |
| Relative difference pre-colonoscopy: Systolic blood pressure (mmHg), median (IQR) | 11.5 (-3.5 - 24.5) | 10.5 (-5.0 - 25.8) | 0.44 | <0.1 |
| Relative difference pre-colonoscopy: Heart rate (bpm), median (IQR) | 5.0 (-1.5 - 11.0) | 3.5 (-5.0 - 12.8) | 0.16 | 0.20 |
| Relative difference post-colonoscopy: Hemoglobin (g/dL), median (IQR) | 0.5 (-1.2 - 1.1) | -0.2 (-1.4 - 1.0) | 0.26 | <0.1 |
| Relative difference post-colonoscopy: Mean corpuscular volume (fL), median (IQR) | -0.4 (-2.2 - 2.6) | -0.7 (-3.3 - 1.4) | 0.27 | <0.1 |
| Relative difference post-colonoscopy: Platelets (10^9/L), median (IQR) | 19.0 (0.0 - 40.0) | 32.0 (5.0 - 68.0) | 0.09 | <0.001 |
| Relative difference post-colonoscopy: Red cell distribution width (%), median (IQR) | -0.3 (-1.0 - 0.0) | -0.5 (-1.4 - 0.0) | 0.18 | 0.16 |
| Relative difference post-colonoscopy: Creatinine (mg/dL), median (IQR) | 0.2 (-0.1 - 0.3) | 0.1 (0.0 - 0.2) | 0.15 | <0.1 |
| Relative difference post-colonoscopy: Glucose (mg/dL), median (IQR) | 12.5 (-3.5 - 26.8) | 11.5 (-6.5 - 28.0) | 0.38 | <0.001 |
| Relative difference post-colonoscopy: White blood cell count (10^9/L), median (IQR) | 1.0 (-0.2 - 1.6) | 1.7 (-0.0 - 3.0) | 0.05 | <0.001 |
| Relative difference post-colonoscopy: Blood urea nitrogen (mg/dL), median (IQR) | 8.0 (2.5 - 17.0) | 9.0 (3.0 - 15.0) | 0.48 | <0.1 |
| Relative difference post-colonoscopy: Potassium (mmol/L), median (IQR) | 0.3 (-0.1 - 0.6) | 0.3 (0.0 - 0.7) | 0.49 | <0.001 |
| Relative difference post-colonoscopy: Sodium (mmol/L), median (IQR) | -2.0 (-4.0 - 1.0) | -1.0 (-4.0 - 1.0) | 0.41 | <0.001 |
| Relative difference post-colonoscopy: Chloride (mmol/L), median (IQR) | -4.0 (-8.0 - 0.0) | -3.0 (-6.0 - -1.0) | 0.45 | <0.001 |
| Relative difference post-colonoscopy: Diastolic blood pressure (mmHg), median (IQR) | 4.0 (-3.0 - 13.8) | 5.0 (-3.0 - 13.0) | 0.44 | <0.1 |
| Relative difference post-colonoscopy: Systolic blood pressure (mmHg), median (IQR) | 11.5 (-3.5 - 24.5) | 10.5 (-5.0 - 25.8) | 0.44 | <0.1 |
| Relative difference post-colonoscopy: Heart rate (bpm), median (IQR) | 5.0 (-1.5 - 11.0) | 3.5 (-5.0 - 12.8) | 0.16 | 0.20 |
| Hospitalization Characteristics | | | | |
| Admit Source: Self Referral, % (N) | 60.0% (30) | 58.1% (50) | 0.98 | <0.1 |
| Arrived in ED, % (N) | 82.0% (41) | 86.0% (74) | 0.70 | <0.001 |
| Was in an ICU at any point during hospitalization, % (N) | 6.0% (3) | 7.0% (6) | 0.89 | <0.001 |
| Hospitalization occurred during COVID-19 public health advisories, % (N) | 8.0% (4) | 7.0% (6) | 0.90 | <0.1 |
| Discharge Disposition: Home or Self-care, % (N) | 40.0% (20) | 40.7% (35) | 0.92 | <0.001 |
| Number of ICD10s associated with stay, mean (95% CI) | 24.3 (22.8 - 25.8) | 25.1 (24.0 - 26.1) | 0.40 | <0.001 |
| No complications occurred during hospitalization (based on MS-DRG), % (N) | 16.0% (8) | 15.1% (13) | 0.91 | <0.1 |
| Hospitalization related to: Dementia, % (N) | 6.0% (3) | 2.3% (2) | 0.53 | 0.19 |
| Hospitalization related to: GI Bleed, % (N) | 66.0% (33) | 64.0% (55) | 0.96 | <0.1 |
| Hospitalization related to: Cirrhosis, % (N) | 4.0% (2) | 5.8% (5) | 0.95 | <0.001 |
| Hospitalization related to: Diabetes, % (N) | 32.0% (16) | 34.9% (30) | 0.88 | <0.001 |
| Hospitalization related to: Kidney Disease, % (N) | 36.0% (18) | 40.7% (35) | 0.72 | <0.001 |
| Medication ordered before colonoscopy: Antihypertensive, % (N) | 6.0% (3) | 5.8% (5) | 0.74 | <0.1 |
| Medication ordered before colonoscopy: Heparin, % (N) | 4.0% (2) | 4.7% (4) | 0.80 | <0.001 |
| Medication ordered before colonoscopy: Opioid, % (N) | 4.0% (2) | 1.2% (1) | 0.63 | 0.18 |
| Medication ordered before colonoscopy: Tricyclic antidepressant, % (N) | 0.0% (0) | 0.0% (0) | 1.00 | <0.001 |
| Medication ordered before colonoscopy: Warfarin, % (N) | 4.0% (2) | 2.3% (2) | 0.98 | <0.1 |
| Medication ordered after colonoscopy: Antihypertensive, % (N) | 14.0% (7) | 17.4% (15) | 0.78 | <0.001 |
| Medication ordered after colonoscopy: Heparin, % (N) | 4.0% (2) | 9.3% (8) | 0.42 | <0.001 |
| Medication ordered after colonoscopy: Opioid, % (N) | 24.0% (12) | 20.9% (18) | 0.84 | <0.1 |
| Medication ordered after colonoscopy: Tricyclic antidepressant, % (N) | 0.0% (0) | 0.0% (0) | 1.00 | <0.001 |
| Medication ordered after colonoscopy: Warfarin, % (N) | 12.0% (6) | 11.6% (10) | 0.83 | <0.1 |
| Nothing by mouth or liquid diet before colonoscopy, % (N) | 100.0% (50) | 98.8% (85) | <0.001 | 0.15 |
| Number of all blood transfusions given during hospitalization, mean (95% CI) | 5.0 (4.4 - 5.6) | 5.3 (4.8 - 5.8) | 0.45 | <0.001 |
| Number of blood transfusions given before procedure, mean (95% CI) | 0.8 (0.6 - 1.0) | 0.6 (0.5 - 0.8) | 0.28 | 0.11 |
| Number of blood transfusions given after procedure, mean (95% CI) | 1.7 (1.3 - 2.1) | 2.0 (1.7 - 2.3) | 0.28 | <0.001 |
| Number of surgical procedures during hospitalization, mean (95% CI) | 3.0 (2.5 - 3.5) | 2.6 (2.3 - 3.0) | 0.21 | 0.17 |
| Number of surgical procedures between bowel preparation order and colonoscopy, mean (95% CI) | 0.0 (-1) | 0.0 (-1) | 1.00 | <0.001 |
| Number of surgical procedures after colonoscopy, mean (95% CI) | 0.4 (0.2 - 0.6) | 0.5 (0.3 - 0.6) | 0.57 | <0.001 |
| Colonoscopy Characteristics | | | | |
| Colonoscopy performed before noon (12 PM), % (N) | 34.0% (17) | 36.0% (31) | 0.96 | <0.001 |
| Same-day double exam: Colonoscopy and esophagogastroduodenoscopy, % (N) | 50.0% (25) | 55.8% (48) | 0.63 | <0.001 |
| Same-day double exam: Colonoscopy performed with any another endoscopy procedure, % (N) | 4.0% (2) | 3.5% (3) | 0.75 | <0.1 |
| American Society Of Anesthesiologists Rating, mean (95% CI) | 2.4 (1.9 - 2.8) | 2.4 (2.1 - 2.8) | 0.80 | <0.001 |
| Indication: Diarrhea, % (N) | 13.6% (6) | 8.8% (7) | 0.59 | 0.16 |
| Indication: Hematochezia, % (N) | 27.3% (12) | 27.5% (22) | 0.86 | <0.001 |
| Indication: Anemia, % (N) | 25.0% (11) | 31.3% (25) | 0.60 | <0.001 |
| Indication: Abnormal imaging, % (N) | 2.3% (1) | 2.5% (2) | 0.60 | <0.001 |
| Indication: Melena, % (N) | 15.9% (7) | 15.0% (12) | 0.90 | <0.1 |
| Indication: Inflammatory bowel disease, % (N) | 0.0% (0) | 0.0% (0) | 1.00 | <0.001 |
| Indication: Rectal bleeding, % (N) | 2.3% (1) | 2.5% (2) | 0.60 | <0.001 |
| Indication: Abdominal pain, % (N) | 4.5% (2) | 1.3% (1) | 0.60 | 0.20 |
| Indication: Other, % (N) | 11.4% (5) | 11.3% (9) | 0.78 | <0.1 |

**References**

1. Fox J. Applied regression analysis and generalized linear models: Sage Publications; 2015.

2. fancyimpute · PyPI. [cited 2020 September 15th]; Available from: <https://pypi.org/project/fancyimpute/>
